# Critical Role for Cold Shock Protein YB-1 in Cytokinesis

**DOI:** 10.3390/cancers12092473

**Published:** 2020-09-01

**Authors:** Sunali Mehta, Michael Algie, Tariq Al-Jabry, Cushla McKinney, Srinivasaraghavan Kannan, Chandra S Verma, Weini Ma, Jessie Zhang, Tara K. Bartolec, V. Pragathi Masamsetti, Kim Parker, Luke Henderson, Maree L Gould, Puja Bhatia, Rhodri Harfoot, Megan Chircop, Torsten Kleffmann, Scott B Cohen, Adele G Woolley, Anthony J Cesare, Antony Braithwaite

**Affiliations:** 1 Department of Pathology, University of Otago, 9016 Dunedin, New Zealand; michael.algie@helsinki.fi (M.A.); cushla.mckinney@otago.ac.nz (C.M.); kim.parker@otago.ac.nz (K.P.); luke.henderson@otago.ac.nz (L.H.); maree.gould@otago.ac.nz (M.L.G.); bhatia.puja17@gmail.com (P.B.); rhodri.harfoot@otago.ac.nz (R.H.); adele.woolley@otago.ac.nz (A.G.W.); antony.braithwaite@otago.ac.nz (A.B.); 2 Maurice Wilkins Centre for Biodiscovery, University of Otago, 9016 Dunedin, New Zealand; 3 Centre for Protein Research, Department of Biochemistry, University of Otago, 9054 Dunedin, New Zealand; torsten.kleffmann@otago.ac.nz; 4 Children’s Medical Research Institute, University of Sydney, Westmead, NSW 2145, Australia; tariqaljabry@squ.edu.om (T.S.-J.); ma.weini@singhealth.com.sg (W.M.); jzha5748@uni.sydney.edu.au (J.Z.); tara.bartolec@unsw.edu.au (T.K.B.); pmasamsetti@cmri.org.au (V.P.M.); meganchircop@gmail.com (M.C.); scohen@cmri.org.au (S.B.C.); tcesare@cmri.org.au (A.J.C.); 5 Department of Biomolecular Modelling and Design, Bioinformatics Institute (A*STAR), 30 Biopolis Street, 07-01 Matrix, Singapore 138671, Singapore; raghavk@bii.a-star.edu.sg (S.K.); chandra@bii.a-star.edu.sg (C.S.V.); 6 School of Biological Sciences, Nanyang Technological University, 60 Nanyang Drive, Singapore 637551, Singapore; 7 Department of Biological Sciences, National University of Singapore, 16 Science Drive 4, Singapore 117543, Singapore; 8 Malaghan Institute of Medical Research, 6242 Wellington, New Zealand

**Keywords:** YB-1, cold shock protein, cytokinesis, post-translational modification, phosphorylation, live-cell imaging, confocal microscopy, atomistic modelling

## Abstract

**Simple Summary:**

Y-box-binding protein-1, YB-1, plays an important role in regulating the cell cycle, although precisely how it does the is unknown. Using live cell imaging, we show that YB-1 is essential for initiating the last step of cell division (cytokinesis), required for creation of two daughter cells. Using confocal microscopy we showed that YB-1 regulates the spatial distribution of key proteins essential for cytokinesis to occur and that this required YB-1 to be phosphorylated on several residues. In-silico modeling demonstrated that modifications at these residues resulted in conformational changes in YB-1 protein allowing it to interact with proteins essential for cytokinesis. As many cancers have high levels YB-1 and these are associated with poor prognosis, our data suggest developing small molecule inhibitors to block YB-1 phosphorylation could be a novel approach to cancer therapy.

**Abstract:**

High levels of the cold shock protein Y-box-binding protein-1, YB-1, are tightly correlated with increased cell proliferation and progression. However, the precise mechanism by which YB-1 regulates proliferation is unknown. Here, we found that YB-1 depletion in several cancer cell lines and in immortalized fibroblasts resulted in cytokinesis failure and consequent multinucleation. Rescue experiments indicated that YB-1 was required for completion of cytokinesis. Using confocal imaging we found that YB-1 was essential for orchestrating the spatio-temporal distribution of the microtubules, β-actin and the chromosome passenger complex (CPC) to define the cleavage plane. We show that phosphorylation at six serine residues was essential for cytokinesis, of which novel sites were identified using mass spectrometry. Using atomistic modelling we show how phosphorylation at multiple sites alters YB-1 conformation, allowing it to interact with protein partners. Our results establish phosphorylated YB-1 as a critical regulator of cytokinesis, defining precisely how YB-1 regulates cell division.

## 1. Introduction

Y-box binding protein 1 (YB-1) is a multifunctional member of the cold-shock protein superfamily that plays an important role during development and cancer [1,2]. High levels of *YBX1* mRNA or protein are tightly associated with patient relapse and poor prognosis in multiple cancer types [3,4,5], reviewed in [1]. Fundamental to cancer aggressiveness is uncontrolled sustained cell proliferation [6]. YB-1 is required for continued cell proliferation in vivo, reviewed in [1], and YB-1 overexpression in transgenic mice led to the development of invasive breast cancer in all instances [7]. In addition, reducing YB-1 levels in tumour xenograft models of breast, brain, lung and pharyngeal cancers inhibited cell proliferation [4,8,9]. Furthermore, YB-1 has also been implicated in regulating the proliferation of breast cancer, large B-cell lymphoma, lung adenocarcinoma, neuroblastoma, hepatocellular carcinoma, glioma, renal cell carcinoma and melanoma in vitro [4,10,11,12,13,14,15,16]. YB-1 has been implicated in regulating many genes involved in proliferation [10,11,12,13,14,15] and survival, reviewed in [1,17], including the 70-gene breast cancer “signature” [18] and the E2F family gene cluster [4]. YB-1 has also been shown to disable the p53 pathway to allow continued cellular propagation despite genomic insult [19]. In addition to these studies, overexpression of YB-1 has been shown to result in cell division errors [20]. Consistent with damage tolerance, YB-1 also promotes chemotherapy resistance [12,21,22,23].

In addition to cancer, YB-1 is known to be important during development. Specifically, homozygous deletion of YB-1 in mice results in embryonic lethality due to impaired cell proliferation [24,25] although loss of one allele has no phenotype [25,26]. Knockdown of *ybx1* in zebrafish embryos results in epiboly failure, leading to defects in cell proliferation, pronounced morphological defects and developmental arrest [27]. Thus, functional YB-1 is necessary for proliferation in multiple cell types.

Several lines of evidence suggest that YB-1 functions in the G_1_ phase of the cell cycle. YB-1 regulates cyclin A and cyclin B1 transcription at the G_1_/S phase border [28] and depletion of YB-1 reduced expression of various cyclins and cyclin-dependent kinases and increased expression of checkpoint proteins p21 and p16 [29]. Conversely, ectopic expression of YB-1 increased expression of cyclin D1 and cyclin E [11]. In contrast, a role for an N-terminal 77 amino acid domain of YB-1 has been implicated in regulating progression of the G_2_/M phases of the cell cycle [30] and other studies have demonstrated that inhibiting YB-1 caused an arrest at G_2_/M [31,32]. Furthermore, prolonged exposure of breast cancer cells to YB-1 led to cytokinesis failure and slippage through the G_1_/S border [20]. Thus, it is unclear from these reports whether YB-1 functions in one or more cell cycle phases.

To address this issue, we used live single cell imaging of several cancer cells combined with confocal microscopy to define precisely how YB-1 regulates the cell cycle. Our results show that YB-1 facilitates assembly of microtubules and specification of the cleavage plane in the equatorial region during cytokinesis, the last step in cell division, without affecting other cell cycle phases. This establishes YB-1 as a critical regulator of cytokinesis.

## 2. Results

### 2.1. YB-1 Depletion Causes Cytokinesis Failure, Multinucleation and Accumulation in G_1_

**Figure 1:**
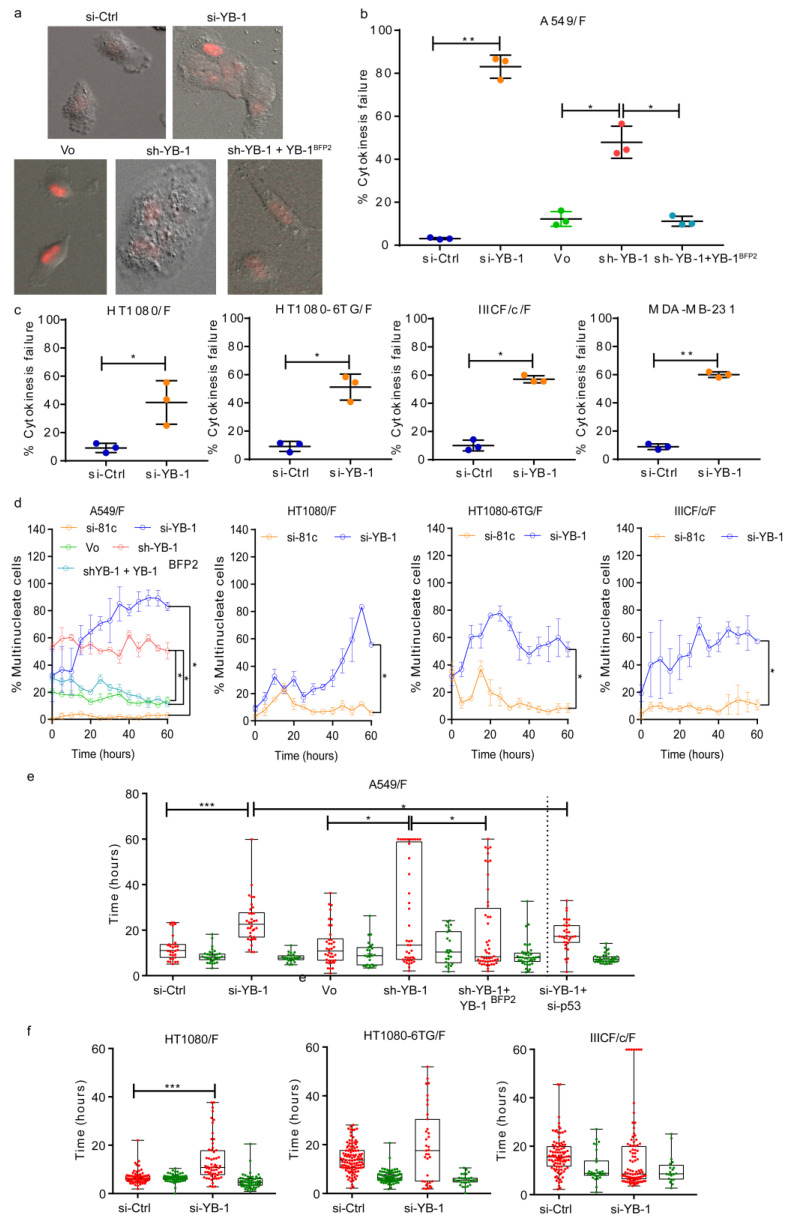
YB-1 depletion inhibits cytokinesis resulting in multinucleation and an increase in G_1_ transit time. (**a**) Examples of A549/F cells treated with control siRNA (si-Ctrl), YB-1 siRNA (si-YB-1), control plasmid (Vo), YB-1 short-hairpin (sh-YB-1) or sh-YB-1 + YB-1^EBFP2^. Images were acquired with a 20× objective. (**b**) Percentage of A549/F cells that fail cytokinesis treated with si-Ctrl, si-YB-1, sh-YB-1, or sh-YB-1 + shRNA-resistant YB-1^EBFP2^. (**c**) Percentage of cells that fail cytokinesis in HT1080/F, HT1080-6TG/F, IIICF/c/F and MDA-MB-231 cells treated with si-Ctrl and si-YB-1. (**b**,**c**). *n* ≥ 100 cells from three independent image fields from one experiment. Dot plots with lines shown mean ± s.d. and one-tailed Student’s t test, * *p* < 0.05, ** *p* < 0.01, ** *p* < 0.001. (**d**) Percentage of multinucleated cells in cultures treated with si-Ctrl, si-YB-1, Vo, sh-YB-1 and sh-YB-1 + YB-1^EBFP2^ 24 h post transfection and followed for up to 80 h. Each point represents the mean ± s.e.m for three independent imaging fields from one experiment. Significance was determined using multiple t-tests with false discovery rate (FDR) correction, * *p* < 0.05. (**e**) Distribution of the G_1_ (red) and S-G_2_-M (green) phases of A549/F cells treated with either si-Ctrl, si-YB-1, si-YB-1 + si-p53, Vo, sh-YB-1 and sh-YB-1+ YB-1^EBFP2^. f. Distribution of G_1_ (red) and S-G_2_-M (green) phases of HT1080/F, HT1080-6TG/F and IIICF/c/F cells treated with either si-Ctrl or si-YB-1. (**e**,**f**) The line in the middle of each box represents the median, the top and bottom outlines of the box represent the first and third quartiles. Significance was determined using the Mann–Whitney U test, * *p* < 0.05, ** *p* < 0.01, *** *p* < 0.001 and **** *p* < 0.0001.

To investigate YB-1 function in the cell cycle, live cells were tracked using time lapse imaging after YB-1 depletion. Five cell lines were used, including 4 transduced with the FUCCI (F) cell cycle reporter constructs [33]: A549/F lung cancer cells with wild-type (wt) *TP53* [34]; HT1080/F fibrosarcoma cells with wt*TP53* [35] and the variant HT1080-6TG (6TG/F) with mutant *TP53* [36]; the spontaneously immortalized IIICF/c/F skin fibroblast line from a Li Fraumeni patient [37]; and MDA-MB-231 breast cancer cells with mutant *TP53*. Cells were transfected with control (si-Ctrl) or YB-1 siRNA (si-YB-1) and Western blotted to confirm YB-1 depletion (Figure S1a). Live-cell imaging was started 24 h post siRNA transfection with images collected using differential interference contrast (DIC) bright field and fluorescent microscopy every 6 min for 60 h. In all cell lines tested, YB-1 depletion induced a striking phenotype of disrupted cytokinesis (Figure 1a–c). Live cell imaging showed that 50–80% of mitoses in YB-1 siRNA-treated cultures failed to complete cytokinesis, compared to approximately 10% in the controls (Figure 1b,c). Transfection of an shRNA targeting endogenous YB-1 3′-UTR (sh-YB-1) also depleted YB-1 and induced cytokinesis failure (Figure 1b). To test if overexpression of YB-1 can rescue this phenotype, we co-transfected cells with a shRNA targeting the 3′-UTR of endogenous YB-1 and with a plasmid overexpressing the YB-1^EBFP2^ fusion protein which is not affected by sh-YB-1. Results show that cytokinesis failure was rescued by co-transfection with a YB-1^EBFP2^ (Figure 1b), confirming that this phenotype is specifically due to loss of YB-1. As expected, cytokinesis failure resulted in significant accumulation of multinucleated cells over time (Figure 1d, Videos S1 and S2) and overexpression of YB-1^EBFP2^ along with sh-YB-1 significantly decreased the number of multinucleated cells (Figure 1d). Finally, depletion of YB-1 resulted in reduced cell proliferation (Figure S3a). Of interest, YB-1 depletion also increased the G_1_ duration of the nuclei in (multinucleated) A549/F and HT1080/F cells, but not in the nuclei of 6TG/F or IIICF/c/F cells (Figure 1e,f) and co-expression of YB-1^EBFP2^ along with YB1 sh-RNA reversed this phenotype (Figure 1e). To test if this was p53 dependent, A549/F cells were co-transfected with siRNAs against p53 (si-p53) and YB-1. Co-depletion of YB-1 and p53 (Figure S1a,c) significantly decreased G_1_ duration compared to depletion of YB-1 alone (Figure 1e). Collectively, the above results show that YB-1 regulates cytokinesis and in turn cell division independently of p53, but causes an increase in G_1_ duration of the daughter nuclei, which is p53-dependent.

### 2.2. YB-1 Is Required for Initiation of Cleavage Furrow Formation

Cytokinesis is the final step during cell division required for formation of two daughter cells [38]. Cytokinesis begins after chromosome segregation, followed by defining the cleavage plane resulting in formation of a cleavage furrow, ingression and abscission, which ends with the physical separation of the daughter cells [38]. Cleavage furrow ingression is controlled by proteins involved in regulating spindle assembly dynamics, which include microtubule associated proteins (α-tubulin), the centraspindlin complex, and the chromosome passenger complex (CPC), comprised of Survivin, Borealin, Inner Centromere Protein (INCENP), and Aurora B kinase (AURKB) [39]. One of the key requirements for completion of cytokinesis is the assembly of the microtubules, specification of the cleavage plane and timely localization of the CPC to the spindle mid-zone [38,39]. Accumulation of the CPC from the spindle midzone to the equatorial cortex, while maintaining the microtubule assembly are required for defining the cleavage plane. Continued localization of the CPC along with timely accumulation of the actin filaments at the cleavage plane to form the actinomyosin ring are required for sustained cleavage furrow ingression [39,40]. Defects in any of these processes will result in cytokinesis failure.

**Figure 2:**
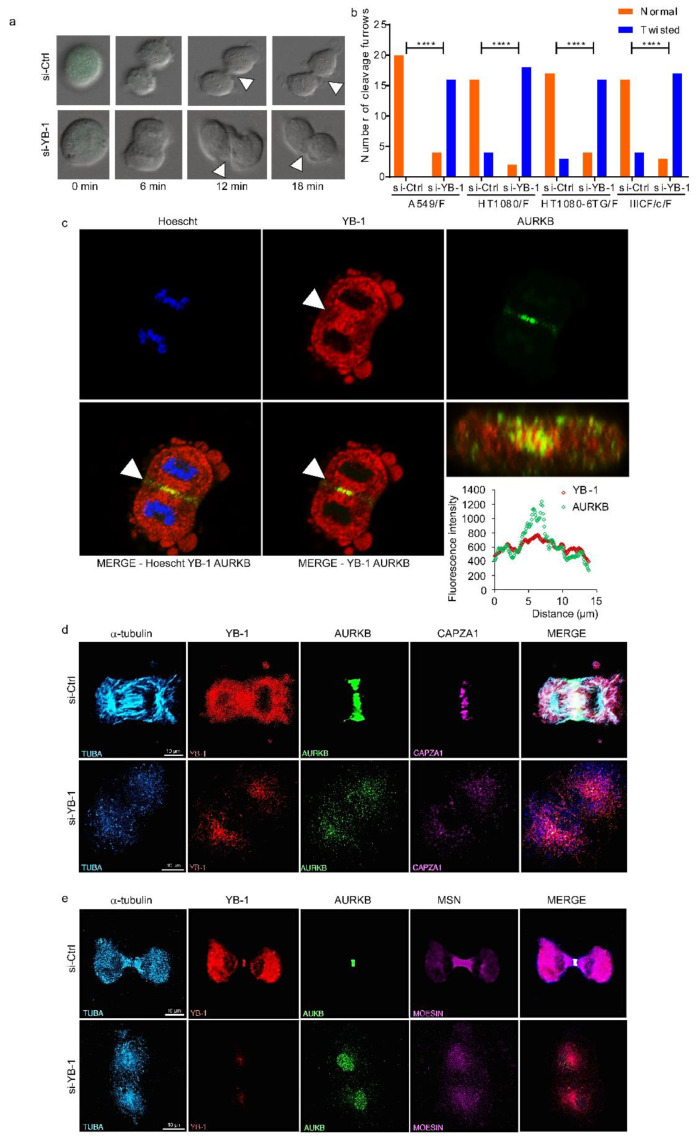
YB-1 depletion results in failure of Chromosome Passenger Complex (CPC) recruitment to the cleavage furrow. (**a**) Examples from live cell imaging of an A549/F cell treated with si-Ctrl and forming a stable cleavage furrow (left panel) or si-YB-1 and forming a twisted cleavage furrow (right panel). Images were acquired with a 20× objective. (**b**) Quantitation of normal versus twisted cleavage furrows in A549/F, HT1080/F, 6TG/F and IIICF/c/F cells treated with either control siRNA (si-Ctrl) or targeting YB-1 (si-YB-1). Twenty cells were counted for each condition. Significance was determined using Chi-Square and Fisher’s Exact test. *p* < 0.05 was considered to be significant. **** *p* < 0.0001. (**c**) Enrichment of YB-1 at the cleavage furrow in A549 cells. (**d**,**e**) A549 cells were treated with either an si-Ctrl (top row) or an si-YB-1 (bottom row) and subsequently immunostained with antibodies against (from left to right) α-tubulin (cyan), YB-1 (red), AURKB (green), CAPZA1 or MSN (magenta) together with a merged image of all four. (**d**) Accumulation of CAPZA1 to the cleavage furrow and (**e**) accumulation of MSN to the cleavage furrow. (**c**–**e**) Images were acquired using a 60x oil-immersion objective.

To define which cytokinesis stage is affected by YB-1 depletion we analysed our live cell imaging data, focusing on phenotypes during late mitosis and cytokinesis (Videos S1 and S2). DIC live cell imaging revealed that cells with reduced YB-1 form a ‘twisted’ cleavage furrow (Figure 2a, see arrows) at a significantly higher frequency than those treated with the si-Ctrl (Figure 2b), thus preventing ingression and consequently cytokinesis. This suggests that YB-1 is required for formation of the cleavage furrow. To test this, we first used confocal microscopy to determine whether YB-1 is localized at or near the cleavage furrow, using a validated, highly specific antibody to the N-terminus of YB-1 [5,41,42] (and Figure S1a,b). Results (Figure 2c) show that as expected AURKB (a marker of CPC) is enriched at the spindle mid-zone. YB-1 is also localized at the spindle mid-zone and appears to intercalate within AURKB. To understand better how YB-1 functions during ingression, we identified 271 proteins from A549 and 280 proteins from MDA-MB-231 cells bound to YB-1 using immunoprecipitation (IP) without cross-linking followed by LC-MS/MS (Figure S2a). We also identified 461 proteins from MDA-MB-231 cells bound to YB-1 using IP with cross-linking followed by LC-MS/MS. Functional analysis using Pantherdb [43] showed an enrichment for proteins involved specifically in cytoskeleton organization, cell cycle and cytokinesis (Figure S2b,c). Using a combination of candidate and database approach we identified specific YB-1 protein partners involved in cytokinesis, which included tubulins (TUBB, TUBB4B and TUBA1B), β-actin (ACTB), the actin-related protein moesin (MSN) and the non-motor actin binding protein CAPZA1, supporting a role for YB-1 in ingression. Next, additional confocal microscopy was carried out which included these proteins. Samples were collected at 48 h after treating cells with either si-Ctrl or si-YB-1. In control cells, YB-1 was again enriched at the spindle mid-zone (Figure 2d) and also at the ingression furrow (Figure 2e), where it aligned with the microtubules (α-tubulin) (Figure 2d,e). AURKB (a marker of the CPC complex) localized at the actinomyosin ring, while CAPZA1 and MSN localized at the actinomyosin ring (Figure 2d) and at the ingression furrow (Figure 2e) respectively. In YB-1 depleted cells, α-tubulin failed to organize and assemble to form the spindle mid-zone, while the other three proteins failed to localize to the actinomyosin ring, remaining disperse throughout the daughter nuclei (Figure 2d,e). The lack of assembly of the spindle mid-zone formation was not due to reduced protein levels (Figure S1d). Taken together, these results suggest that YB-1 plays an essential role in facilitating assembly of the microtubules (critical for specification of the cleavage plane) followed by localization of the CPC at the spindle mid-zone. Lack of microtubule assembly in YB-1 depleted cells is consistent with reports in the literature [20,44].

**Figure 3:**
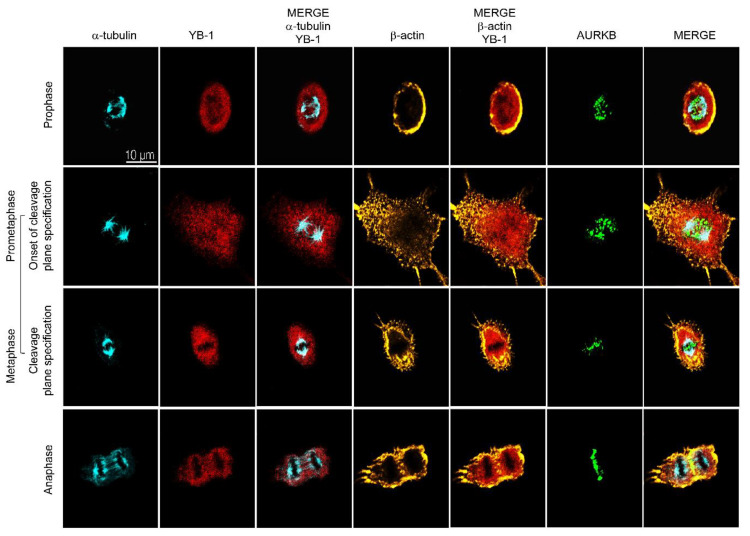
YB-1 is required for formation of the cleavage furrow. Examples of synchronized A549 cells enriched for cytokinesis, immunostained with antibodies against (from left to right) α-tubulin (cyan), YB-1 (red), merge of α-tubulin and YB-1, β-actin (yellow), merge of β-actin and YB-1, AURKB (green) followed by a merged image of all three. Images were acquired using a 60× oil-immersion objective.

To determine how YB-1 contributes to establishment of the cleavage plane, we synchronized A549 cells at G_1_ using a double thymidine block, released the block and once the cells had reached mitosis (7 h later) fixed them sequentially at 5 min intervals for up to 90 min to capture those progressing towards cytokinesis. These cells were then stained for YB-1, α-tubulin (microtubules), β-actin and AURKB and imaged using confocal microscopy. Results show that during prophase YB-1 forms a ring encapsulating AURKB and the microtubules (α-tubulin), with β-actin forming a corona around the cell membrane (Figure 3, top row). The YB-1 ring is maintained and appears to shield the segregating chromosomes during prometaphase (Figure 3, row 2) and metaphase (Figure 3, row 3) as the microtubules align at the two poles and AURKB accumulates from the centre of the spindle mid-zone to the equatorial cortex so defining the position of the cleavage plane. At anaphase the cleavage plane has matured, YB-1 is present at the mid-zone and also appears perinuclear surrounding the microtubule fibres in the emerging daughter cells. YB-1 also appears to intercalate within the actinomyosin ring formed by AURKB, β-actin (Figure 3, bottom row) and other CPC proteins. These results show that YB-1 is intimately associated with proteins required to form the cleavage plane and in conjunction with the data in Figure 2, suggest that YB-1 facilitates the spatio-temporal organization of the microtubules, which in turn is responsible for organization of the CPC and β-actin at the spindle midzone.

### 2.3. YB-1 Depletion Causes Cytokinesis Failure in Zebrafish Embryos Resulting in Developmental Defects

**Figure 4:**
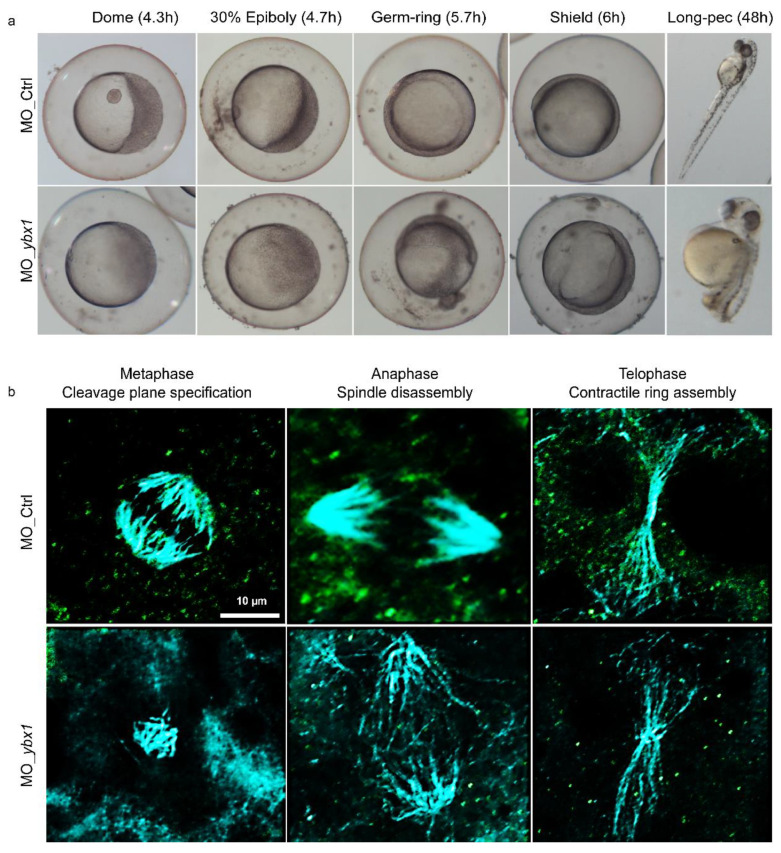
Ybx1 depletion results in developmental defects due to cytokinesis failure in zebrafish embryos. (**a**) Examples of zebrafish embryos at 4.3, 4.7, 5.7, 6 and 48 h post fertilization treated 4 ng of either control morpholino (MO_Ctrl, top panel) or a morpholino targeting *ybx1* (MO_*ybx1*, bottom panel). Images were acquired using 10× objective. (**b**) Examples of zebrafish embryonic cells that have been treated with 4 ng of MO_Ctrl (top panel) or MO_*ybx1* (bottom panel) 5.7–6 h post-fertilization undergoing cytokinesis, immunostained with antibodies to Ybx1 (green) and α-tubulin (cyan). Images were acquired using a 60× oil-immersion objective.

Zebrafish Ybx1 protein has been shown to be essential for development and either mutation or depletion of zebrafish Ybx1 resulted in failure to initiate epiboly, reduced cell proliferation and eventually developmental arrest [27,45,46,47]. To determine if the cause of the developmental defects could be due to cytokinesis failure, we injected morpholino-oligonucleotides (MO) targeted to *ybx1* or a non-targeting control, at the one or two cell stage and characterized the effects using DIC and confocal microscopy. DIC images of embryos treated with MO_*ybx1* showed failure to initiate epiboly due to impaired cell proliferation compared to the control (Figure 4a) as had been previously reported [27]. Moreover, MO_*ybx1* treated embryos resulted in developmentally defective fish 48 h post-fertilization (Figure 4a, Figure S1e). Next, to establish if failure of epiboly initiation in *ybx1*-depleted embryos is due to a disruption in the organization of microtubules, we performed whole-mount immunofluorescence for Ybx1 and α-tubulin and images were acquired by confocal microscopy. Results show that in MO_*ybx1* treated embryos there was a failure of microtubule organization and assembly compared to the controls, consistent with our findings in cell culture (Figure 2 and Figure 3). This leads to a failure in cleavage plane specification (Figure 4b). The results suggest that YB-1 is essential for regulating the process of cytokinesis during development.

### 2.4. p90 Ribosomal-S6 Kinase (RSK) Phosphorylates YB-1 and Is Required for Cytokinesis.

YB-1 function is thought to be regulated through phosphorylation [42]. Inhibiting YB-1 phosphorylation on serine 102 with a mutation to alanine (S102A) prevented cell proliferation [48,49] and transactivation of various genes including *EGFR* [50] and *PIK3CA* [51] and *NOTCH4* [52]. Protein Kinase B (AKT) [48], p90 S6 Ribosomal Kinase (RSK) [53] and Extracellular Signal Regulated Kinase (ERK) [49,54] have all been implicated in YB-1 S102 phosphorylation, and inhibiting RSK suppressed the ability of YB-1 to promote proliferation [53,55]. Inhibition of RSK/YB-1 signalling has shown to improve sensitivity of prostate cancer cells treated with enzalutamide [56]. To identify putative kinases capable of phosphorylating YB-1 at either S102 or at other sites on the protein, we used the web server for Group-based Prediction System 2.1. Xue et al. developed an algorithm to predict kinase specific phosphorylation sites using a motif length selection approach [57]. This identified four candidate kinases: AKT [48], RSK [53], Casein kinase 2 (CK2) [58] and AURKB.

**Figure 5:**
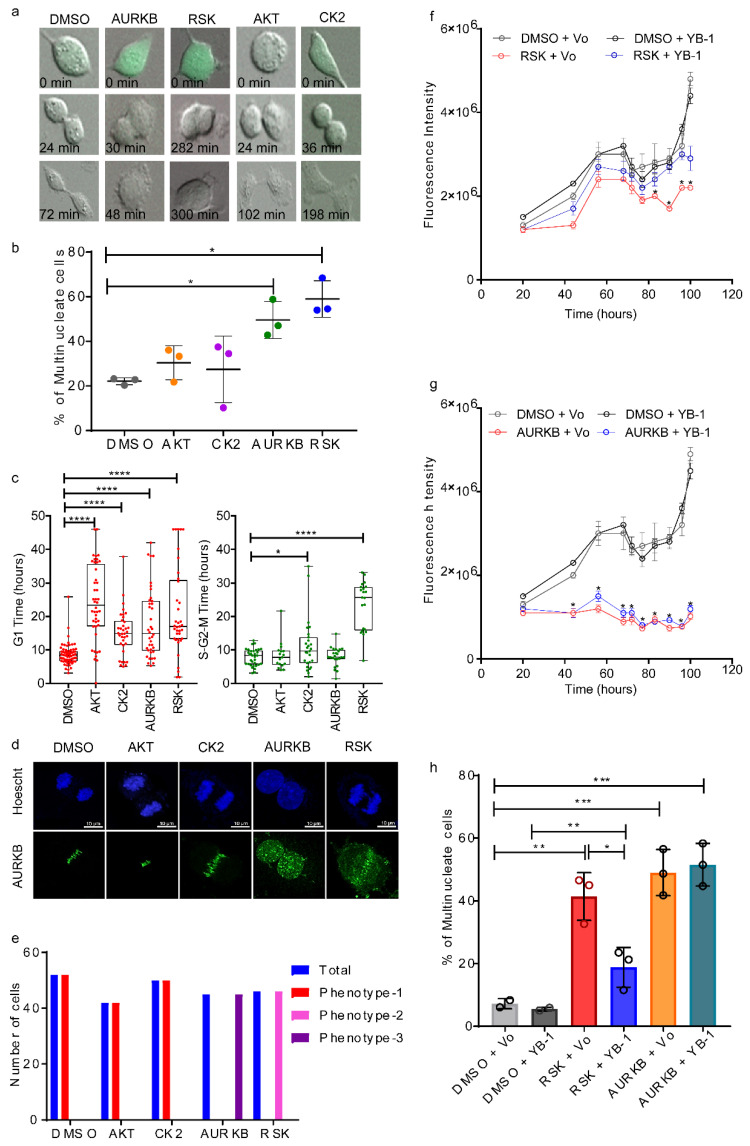
RSK inhibition phenocopies YB-1 depletion. Results from cells treated with solvent control (DMSO), Barasertib-1µM (AURKB), BI-D1870-5µM (RSK), MK-2206HCl-10µM (AKT) or Silmitasertib-10µM (CK2). (**a**) Examples of failed cytokinesis after treating A549/F cells. Images were collected using a 20x objective. (**b**) Percentage of multinucleate A549/F cells 40 h post treatment. *n* ≥ 100 cells, three independent image fields, one experiment. Dot plots with lines shown mean ± sd., one tailed student t test, * *p* < 0.05. (**c**) Distribution of time in G_1_ (left) and in S-G_2_-M phases (right) of A549/F cells post treatment, Mann–Whitney U test, * *p* < 0.05 and **** *p* < 0.0001. (**d**) Example of A549 cells post treatment undergoing cytokinesis stained with Hoechst (blue) or anti-AURKB (green). Images were acquired using a 60× oil-immersion objective. (**e**) Quantitation of AURKB staining at the cleavage furrow in A549 cells; Phenotype1: tightly localized; Phenotype 2: diffuse; Phenotype 3: diffuse nuclear staining. (**f**,**g**) The amount of SYBR Green I fluorescence over time in cells transfected with Vo or YB-1^EBFP2^ and treated with DMSO or BI-D1870 (**f**) or Barasertib (**g**) Each point represents the mean ± s.e.m in ≥ 3 replicates, Student’s t-tests * *p* < 0.05. (**h**) Percentage of multinucleate cells in A549/F cells treated with 200 ng of Vo or YB-1 and either DMSO, BI-D1870 or Barasertib for 40 h, one-way ANOVA with Tukey’s multiple comparison test. * *p* < 0.05, ** *p* < 0.01, *** *p* < 0.001.

To determine whether inhibiting putative YB-1 regulators affected cytokinesis, A549/F cells were treated over several days with solvent (DMSO), or different concentrations of Barasertib (AURKB inhibitor), BI-D1870 (RSK inhibitor), MK-2206HCl (AKT inhibitor) or Silmitasertib (CK2 inhibitor). Cultures were then visualized using an IncuCyte live imaging system and cell proliferation analysed to determine a non-cytotoxic dose for each compound (Figure S3b). A549/F cells were then treated with the various inhibitors at the determined concentrations and live cell imaging was performed as described above, recording images every 6 min for 46 h. Consistent with their known function in promoting cytokinesis, inhibiting AURKB [59] or RSK [60] resulted in cytokinesis failure and multinucleated cells (Figure 5a,b) as was observed with YB-1 depletion. RSK inhibition with BI-D1870 also conferred a delay in cleavage furrow formation (Figure 5a). All four kinase inhibitors resulted in increased G_1_ duration (Figure 5c, left panel), while RSK inhibition also resulted in an approximate three-fold increase in S-G_2_-M duration (Figure 5c, right panel). A small, but significant, S-G_2_-M duration increase was also observed with the CK2 inhibitor.

**Figure 6:**
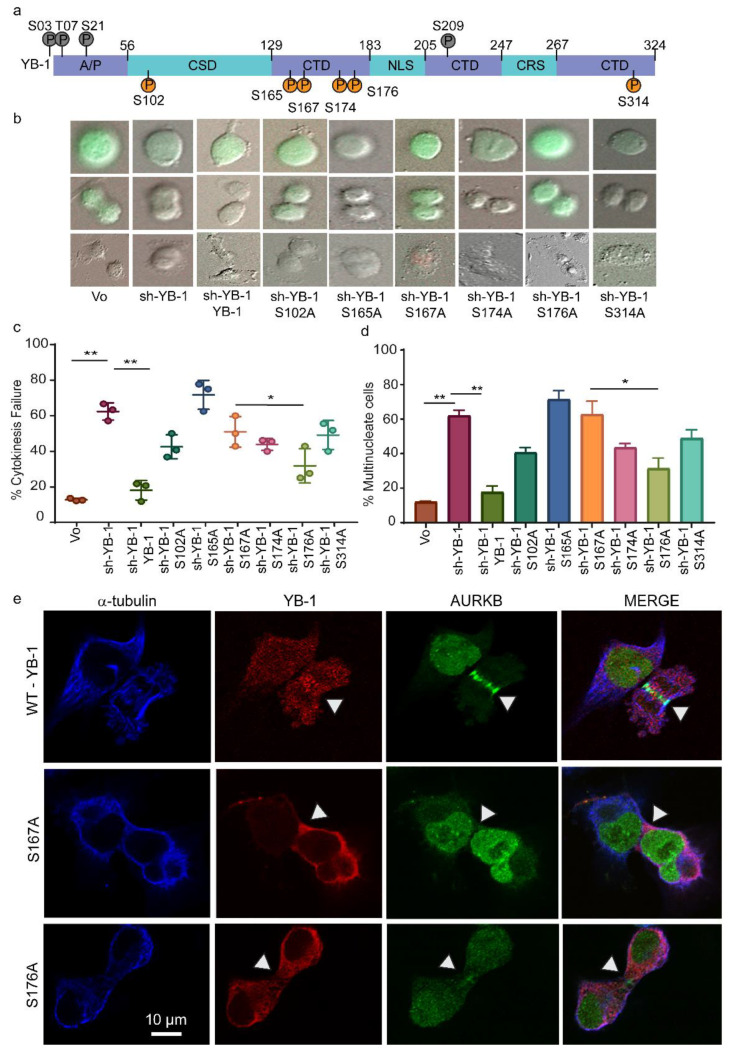
Phosphorylation defective YB-1 mutants cannot rescue cytokinesis failure following YB-1 depletion. (**a**) Schematic showing the YB-1 protein and the location of the phosphorylation sites detected in MDA-MB-231, A549 and T47D cells (Common: orange circles; summarized in Table 1, and rare: grey circles). A/P: Alanine/proline-rich domain; CSD: Cold shock domain; CTD: C–terminal domain; NLS: Nuclear localization signal; and CRS: Cytoplasmic retention signal. (**b**–**e**) Data collected from A549/F cells treated with either Vo or sh-YB-1 in combination with Vo or YB-1^EBFP2^ or the six phosphorylation defective mutants (S102A, S165A, S167A, S174A, S176A and S314A). (**b**) Examples of A549/F cells undergoing cytokinesis defects. Images were acquired using a 20× objective. (**c**) Quantitation of the experiment shown in (**b**). Each point represents the mean ± s.e.m. for ≥ 3 independent image fields from one experiment. Significance was determined using one-way ANOVA followed by Tukeys multiple comparison test. * *p* < 0.05, ** *p* < 0.01. (**d**) The percentage of multinucleated cells 64 h post treatment with Vo or sh-YB-1 in combination with Vo or YB-1 or the six phosphorylation defective mutants (S102A, S165A, S167A, S174A, S176A and S314A). Each point represents the mean ± s.e.m for eight imaging fields from one experiment. Significance was determined using multiple t-tests with FDR correction, * *p* < 0.05. (**e**) Examples of CPC staining in A549 cells after treatment with sh-YB1 in combination with either YB-1, S167A or S176A mutants. Cells are stained (left to right) with antibodies to α-tubulin (blue), YB-1 (red) or AURKB (green) followed by merged images. Images were acquired using a 60× oil-immersion objective.

We then assessed how kinase inhibition affected cytokinesis and CPC localization using AURKB as a marker. Control, CK2 and AKT inhibition resulted in normal CPC enrichment at the equatorial cortex (phenotype-1, Figure 5d,e). However, with RSK inhibition, CPC only partially enriched at the center of the spindle mid-zone and also appeared punctate throughout the nucleus (Phenotype-2, Figure 5d,e). As expected, no cleavage furrow formed in cells treated with Barasertib, where the CPC remained in the nucleus, again appearing punctate (Phenotype-3, Figure 5d,e). To confirm if the cytokinesis failure observed by RSK inhibition was due to reduced YB-1 phosphorylation, we performed Western blot analysis using an antibody that can detect phosphorylation of YB-1 at S102. Consistent with RSK-dependent regulation of S102, we observed YB-1 phosphorylation at S102 was reduced in cells treated with a RSK inhibitor but not with an AURKB inhibitor (Figure S1f,g). In agreement, we found that over-expressing YB1^EBFP2^ partially rescued the proliferation and multinucleation phenotypes induced with RSK inhibition (Figure 5f,h) but there was no rescue after AURKB inhibition (Figure 5g,h). Furthermore, the phosphorylation defective S102A mutant failed to rescue from the multinucleation phenotype following YB-1 depletion (Figure 6b–d). Cumulatively, of the kinases tested, only inhibiting RSK reduced YB-1 phosphorylation and phenocopied cytokinesis failure observed with YB-1 depletion. This suggests RSK regulates YB-1 function during cytokinesis

### 2.5. Phosphorylation Defective YB-1 Mutants Fail to Complete Cytokinesis.

In addition to phosphorylation at S102, phosphorylated serines at residue 165 (pS165) and 176 (pS176) were recently identified by mass spectrometry [61,62]. Mutating these residues impaired the ability of YB-1 to transactivate the NFκB promoter, reduced cell proliferation, and impacted tumour progression [61,62]. To determine which phosphorylation sites on YB-1 are important for cytokinesis, we immunopurified YB-1 from three cell lines (A549 and two breast cancer lines; MDA-MB-231 and T47D). YB-1 was then electrophoretically separated by SDS-PAGE, excised, digested with trypsin, and phosphorylated peptides enriched with TiO_2_ were then analysed using global LC-MS/MS profiling. We identified 10 phosphorylated residues (Figure S4a and Table 1), five of which were identified in all three cell lines in multiple repeat experiments, including pS165 and pS176 and novel phosphorylated residues pS167, pS174 and pS314 (Table 1, Figure 6a). Of note, the most frequently studied (pS102) residue appeared to be difficult to detect in LC-MS/MS as it was observed in only 3/8 runs and in only one cell line (Table 1, Figure 6a). Of interest, all the common phosphorylated residues and pS102 are conserved in higher mammalian species and none of these six sites is mutated in 7799 tumours across 32 different tumour types analysed using The Cancer Genome Atlas [63] data from cBioPortal [64]. Thus, phosphorylation of these residues may be critical for YB-1 function.

To determine whether phosphorylation of these conserved residues contributes to YB-1 function in cytokinesis, we created YB-1^EBFP2^ expression constructs with an alanine substitution at each phosphorylated serine. A549/F cells were transfected with sh-YB-1 with or without co-expression of YB-1^EBFP2^ or phospho-defective mutants S102A, S165A, S167A, S174A, S176A and S314A. Cultures were imaged and analysed as described above. The mean blue fluorescence intensity was used as a marker for expression of the plasmids which showed that all exogenous mutant proteins were expressed at similar levels (Figure S4c). Interestingly, all 6 phosphorylation defective mutants failed to rescue the cytokinesis defects induced by depleting endogenous YB-1, although S176A was attenuated (Figure 6b–d). To determine how YB-1 phosphorylation affected CPC recruitment during cytokinesis, we co-expressed sh-YB-1 with S167A or S176A and performed immunofluorescence. Cells co-expressing the sh-YB-1 and S167A failed to form the cleavage furrow due to a failure in organization of the microtubules and localization of the CPC to the spindle mid-zone (Figure 6e, middle panel), while S176A still enabled partial cleavage furrow formation (Figure 6e, bottom panel). These results suggest that phosphorylation of YB-1 is essential for its function during cytokinesis. To test, if RSK phosphorylates YB-1 at other residues in addition to S102, we carried out targeted mass spectrometry after RSK inhibition. Results showed reduced phosphorylation of YB-1 at S165, S167, S174 and S176 after RSK inhibition (Figure S4b). Moreover, over-expressing YB-1^EBFP2^ partially rescued the proliferation and multinucleation phenotypes induced with RSK inhibition (Figure 5f–h). Taken together, these results suggest that RSK phosphorylates YB-1 at these five sites and is essential for YB-1 function during cytokinesis.

**Figure 7:**
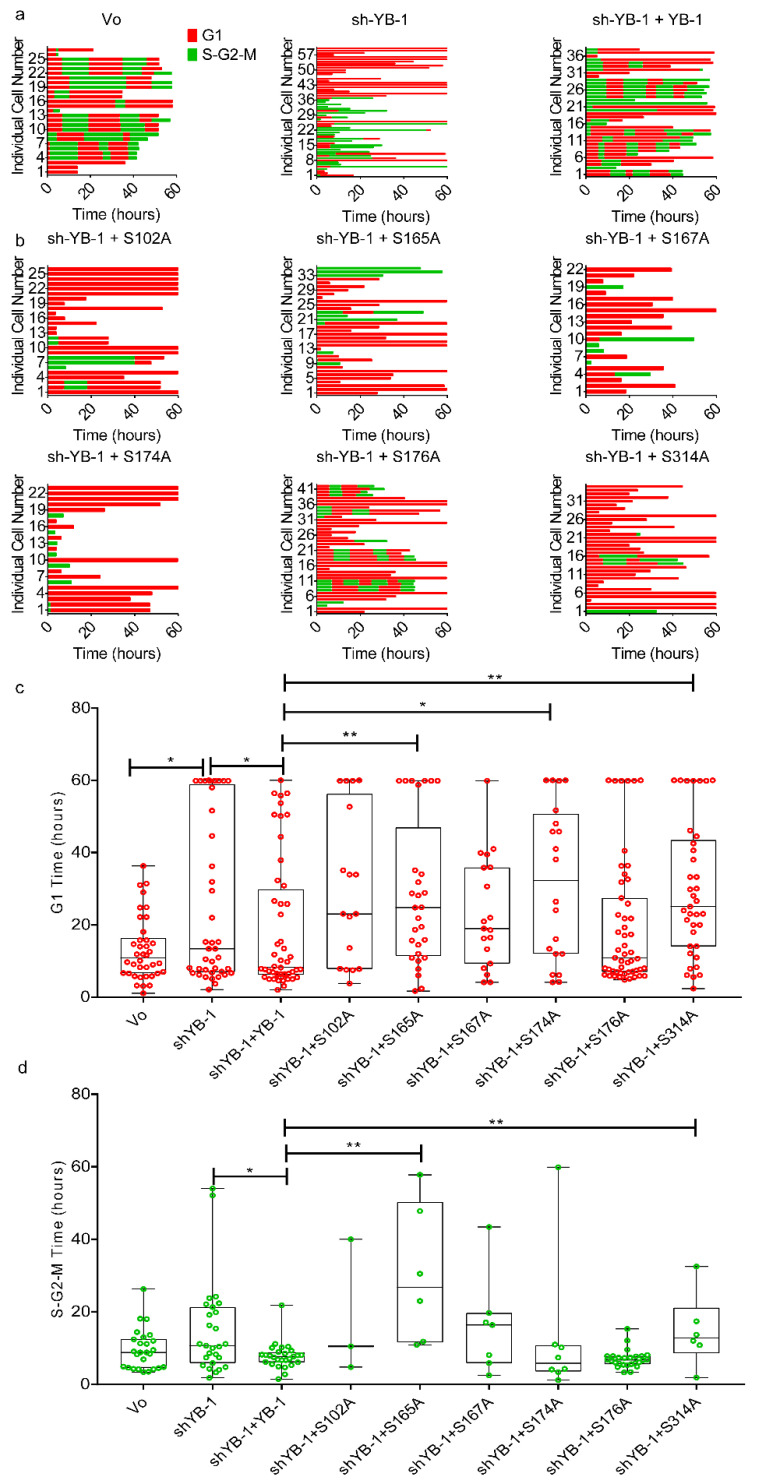
Phosphorylation defective YB-1 mutants do not rescue from cytokinesis failure. (**a**,**b**) Shows the starting phase of the cell cycle for each tracked A549/F cell using time lapse imaging and quantitates the amount of time spent in each phase of the cell cycle and the number of divisions that each cell undergoes. (**c**) Quantitation of G_1_ phase transit time. (**d**) Quantitation of S-G_2_-M phase transit time. (**c**,**d**) Significance was determined using the Mann–Whitney U test, * *p* < 0.05, ** *p* < 0.01, *** *p* < 0.001 and **** *p* < 0.0001.

To determine the impact of phosphorylated YB-1 on other cell cycle phases, we quantified G_1_ and S-G_2_-M duration in A549/F cells treated with sh-YB-1 along with YB-1 or phosphorylation defective YB-1 mutants. Cells treated with sh-YB-1 mostly stalled in the G_1_ phase of the cell cycle which was overcome by YB-1^EBFP2^ co-expression, as indicated by alternating red and green (cycling) cells (Figure 7a,c). With the exception of S176A, all mutants were stalled in G_1_ (Figure 7b,c). By contrast, 30% of cells expressing S176A continued to cycle, consistent with this mutant being attenuated. Finally, with the exception of S165A and S314A, none of the mutants increased the time spent in S-G_2_-M phase of the cell cycle (Figure 7b,d). Collectively these data suggest that phosphorylation on all six YB-1 sites is required to complete cytokinesis and progress through the cell cycle.

### 2.6. Cooperative YB-1 Phosphorylation on Multiple residues Alters Protein Folding.

**Figure 8:**
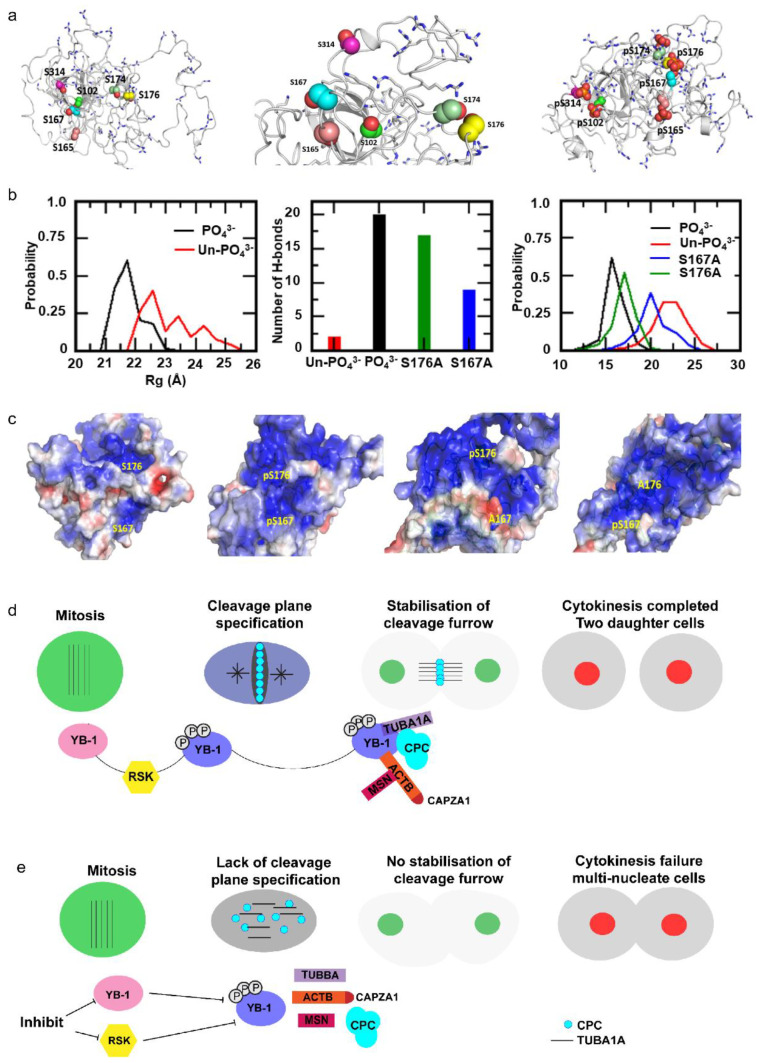
Molecular dynamics simulation models the impact of phosphorylation on localization of the CPC to the cleavage furrow. (**a**) YB-1 model generated using I-TASSER and MD simulations, left: un-phosphorylated, middle: zoomed view of serines and right: phosphorylated states (pYB-1). Spheres represent serines and phosphoserines, thick grey lines represent positively-charged Arg and Lys residues. The ordered cold shock domain is shown at the centre flanked by the disordered long termini. (**b**) Left: Distribution of the radius of gyration (Rg), a measure of protein compaction. Middle: number of hydrogen bonds involving the serines/phosphorylated serines. Right: distribution of the distances between the backbone atoms of residues 167 and 176 sampled during the MD simulations of YB-1 and phosphorylated YB-1. (**c**) Electrostatic surfaces projected on to (left to right) YB1 (unphosphorylated), pYB-1 (phosphorylated), and the two mutants S167A and S176A; the blue and red colours correspond to potentials of +3 kcal/mol and −3 kcal/mol, respectively. (**d**) YB-1 has a disordered structure with neutral and negatively charged pockets. Upon phosphorylation by RSK, YB-1 protein undergoes a structural change, creating a positively charged pocket. We speculate that this pocket interacts with the negatively charged termini of α-tubulin to define the cleavage plane by facilitating the formation of the microtubule structure and localization of the CPC, which, in turn, leads to the stabilization of the cleavage furrow. YB-1 also interacts with β-actin and actin related proteins (CAPZA1 and MSN), promoting cytokinesis. (**e**) When phosphorylation of YB-1 is inhibited or depleted, the cleavage plane defined by the α-tubulin scaffold cannot form resulting in a failure of CPC localization and cytokinesis.

Phosphorylation on multiple residues commonly confers structural rearrangements, regulating protein-protein interactions. To determine how YB-1 phosphorylation affects protein folding, we generated atomic models of YB-1 using the I-TASSER pipeline [65]. The atomic structure of the full length (residues 1–324) YB-1 protein was modelled using the known structure of the cold shock domain (CSD, residues 50-124) [66] with and without phosphorylated residues. The selected template depicts YB-1 to be a disordered protein as has been reported [67]. Based on the model, there are positive charges including arginine and lysine (thick grey lines) both within the CSD and at the N and C termini of YB-1 (Figure 8a). Molecular Dynamics (MD) simulations were carried out to determine structural changes resulting from phosphorylation. Results suggest that phosphorylation causes YB-1 to adopt a fold such that a negatively-charged interior is created, which exposes positively-charged residues at the protein surface. This is anticipated to create a positively charged binding patch capable of interacting with negatively charged protein domains or nucleic acids (Figure 8a–c).

The conformation YB-1 is maintained by hydrogen bonds formed between the phosphorylated residues and other positively charged amino acids, which are reduced in the absence of phosphorylation (Figure 8b,c). Moreover, the number of hydrogen bonds is greatly reduced when S167 cannot be phosphorylated compared to S176, suggesting that S167 has a greater effect on YB-1 conformation compared to S176 (Figure 8b,c). In addition to hydrogen bonds, simulation analyses suggest that phosphorylated YB-1 has a compact structure (lower radius of gyration, Rg) compared to un-phosphorylated YB-1 (Figure 8b,c) and again phosphorylation at S167 has a greater impact on YB-1 conformation than S176 (Figure 8b,c). These modelling predications are consistent with our functional data showing that the S167A mutant had a greater negative impact on cytokinesis than the S176A mutant. Cumulatively, the data indicate that RSK phosphorylates YB-1 on multiple residues, thereby altering YB-1 conformation to facilitate CPC recruitment to the cleavage furrow to promote cytokinesis.

## 3. Discussion

YB-1 has long been known to promote cell proliferation during embryonic development and cancer, as reviewed in [1]. Several reports have suggested that YB-1 regulates the G_1_ to S phase transition whilst others have suggested that YB-1 plays a role at G_2_/M and directly in mitosis [4,28,32]. However, the precise mechanism is unknown. Using live cell imaging and single cell fate tracking we show that YB-1 is critical for completion of cytokinesis in several cancer cell lines and in human fibroblasts. Cells lacking YB-1 traverse the cell cycle without any defect until cytokinesis, where they fail to undergo cytokinesis, resulting in multinucleation. We further demonstrate that over-expression of YB-1 rescues all these defects. Confocal imaging confirmed that the CPC proteins AURKB, CAPZA1 and MSN failed to localize to the cleavage plane after YB-1 depletion. In addition, using synchronized cells and closely spaced time points, imaging data suggest that YB-1 orchestrates the accurate spatio-temporal distribution of the microtubules, actin and CPC to the spindle midzone, thus playing an essential role in defining the cleavage plane. We provide further evidence that zebrafish embryos depleted of Ybx1 fail to assemble and organize the microtubule fibres essential for defining the cleavage plane and completion of cytokinesis. Our results thus provide an explanation for defects in cell proliferation observed in Ybx1-depleted zebrafish embryos [27] and why developmental defects in YB-1 knockout mice are not evident until E11.5–E13.5 [24,25], the time at which there is marked increase in body mass resulting from many cell divisions. Our findings also provide a rationale as to why increased YB-1 levels are often observed in advanced cancers; it likely reflects selection pressures during cancer evolution, assisting in overcoming cytokinesis checkpoints that may be activated during rapid cell division.

YB-1 phosphorylation on S102 is descriptively linked to proliferation and RSK is known to phosphorylate this site [48,53,61,62]. In our study, in addition to S102, we have identified five highly-conserved residues in YB-1 phosphorylated by RSK that are required to facilitate cytokinesis. Mutation of any one of these residues prevented rescue of cytokinesis failure following YB-1 depletion. We also found that these mutants failed to progress through the cell cycle, being ‘stalled’, mainly in G_1_. Using molecular dynamics simulation, we predict that simultaneous phosphorylation of these sites promotes structural alterations in YB-1 protein, resulting in an intense positive potential, which enables YB-1 to interact with the negatively charged termini of α-tubulin [68] and actin filaments [69]. These interactions then allow specification of the cleavage plane and localization of the CPC, CAPZA1 and MSN at the cleavage furrow (Figure 8d). YB-1 depletion, or inhibiting YB-1 phosphorylation results in failure to establish the cleavage plane (Figure 8e).

We note there was a continuum of phenotypic severity in cells expressing the YB-1 phosphorylation defective mutants. We propose these differences reflect the impact that each residue has on promoting YB-1 conformational changes following phosphorylation. For example, our modelling data indicate that when S176 and S174 are phosphorylated, they are located in close proximity to a region with a high concentration of positive charges. Phosphorylation of S174 is therefore likely able to compensate when phosphorylation is blocked at S176, but S165 and S167 are located in a region with a lower concentration of positive charges, thus it is unlikely that pS165 is able to effectively compensate for lack of phosphorylation at S167. In agreement, the S167A mutation induced more severe phenotypes than the S176A mutation. Thus, we provide evidence that simultaneous post-translational modification of YB-1 is required to promote its cytokinesis functions.

A previous study [20] identified cell division errors following massive over-expression of YB-1 from an inducible promoter. In contrast, we show conclusively through depletion and rescue experiments that YB-1 is a positive regulator that promotes cytokinesis, and in the absence of well-regulated YB-1 function, cytokinesis failure ensues. We anticipate that massive over-expression of YB-1 as used in the above paper, de-regulates the homeostasis within the cytokinesis regulatory network, thereby artificially inducing cytokinesis failure.

In addition to cytokinesis failure, YB-1 depletion caused an increase in G_1_ transit time of the daughter nuclei of wild-type p53 cells that was counteracted by p53 depletion, suggesting the arrest is p53 dependent. p53 activation following cytokinesis failure has been shown to result from activation of the PIDDosome complex or the Hippo pathway in response to supernumerary chromosomes [70,71]. Both pathways lead to p53 stabilization by inhibiting or degrading MDM2, the E3 ligase that promotes p53 degradation. Thus, it is likely that one or both of these pathways are activated when YB-1 function is impaired. However, with the exception of S176A, the other phosphorylation defective YB-1 mutants exhibited a greater frequency of cells stalled in G_1_ compared with YB-1 shRNA alone. This suggests the G_1_ arrest may not be due solely to the presence of supernumerary chromosomes, but may involve direct interaction of YB-1 with either MDM2 or p53. We anticipate correctly phosphorylated YB-1 can over-ride the checkpoint, possibly by binding directly to p53 [72], suggesting that when YB-1 phosphorylation is impaired, the checkpoint remains activated.

## 4. Materials and Methods

### 4.1. Cell Culture

A549 (human lung adenocarcinoma), HT1080 and HT1080-6TG [36] (human fibrosarcoma), MDA-MB-231(triple negative breast cancer) and IIICF/c (skin fibroblast cell line from Li Fraumeni patient [37]) cells were cultured in Dulbecco’s Modified Eagle Medium (DMEM, Life Technologies, Carlsbad, CA, USA or Sigma-Aldrich, St. Louis, Mo, USA) supplemented with 10% foetal bovine serum (FBS), 1% Non-essential amino acids (NEAA) and 1% GlutaMax in a 37 °C humidified incubator at 5% CO_2_ unless stated otherwise. Antibiotics were not used. All cell lines were validated for authenticity by CellBank Australia (http://www.cellbankaustralia.com/) using STR profiling. All cell lines are regularly tested negative for mycoplasma contamination.

### 4.2. Generation of FUCCI Cell Lines

FUCCI (Fluorescent Ubiquitination-based Cell Cycle Indicator) was used to identify cell cycle phases during live cell imaging at a single cell resolution [73]. The FUCCI cells consist of red and green fluorescent protein fused to cell cycle regulators: Cdt1 and Geminin. During the cell cycle, these two proteins are ubiquitinated by specific ubiquitin E3 ligases, which targets them for proteasomal degradation [73]. In the G_1_ phase of the cell cycle, Geminin is degraded, therefore, only Cdt1 tagged with red fluorescent protein is present, resulting in red cells. In the S, G_2_ and M phases of the cell cycle, Cdt1 is degraded and only Geminin tagged with green fluorescent protein is present, resulting in green cells. During the G_1_/S transition there is decreasing amounts of Cdt1 and increasing amounts of Geminin, so when both proteins are present the cells appear yellow [73]. mVenus-hGeminin(1/110)/pCSII-EF and mCherry-hCdt1(30/120)/pCSII-EF (a kind gift from Atsushi Miyawaki and Hiroyuki Miyoshi) were individually packaged into lentivectors using the 2 nd generation packaging system, and the viral supernatants were used simultaneously to co-infect target cells. Three days post-transduction, cell cultures were sorted at the Westmead Institute for Medical Research (WIMR) flow cytometry core (Sydney, Australia) for mVenus fluorescence, allowed to expand for 5–7 days, and sorted again for mCherry fluorescence. Proper progression of red/green coloration during cell cycling was confirmed with live cell imaging as described below before use. FUCCI expressing cell lines used include A549/F, HT1080/F, HT1080-6TG/F and IIICF/c/F.

### 4.3. siRNA Transfection

Cells were reverse transfected with stealth modified 25 bp duplex siRNA targeted to YB-1 (si-YB-1 5′-GGUCCUCCACGCAAUUACCAGCAAA-3′) [4] and a control siRNA (si-Ctrl 5′-CCACACGAGUCUUACCAAGUUGCUU-3′) with no known human mRNA targets [74]. Stealth siRNAs were transfected at a final concentration of 5 nM using Lipofectamine RNAiMax (Invitrogen, Carlsbad, CA, USA). Both siRNAs and RNAiMax were diluted in medium without serum. After 10 min at room temperature, the diluted RNAiMax was added to the siRNAs, and the mixture was incubated for a further 15 min. The lipoplexes formed were added to cells. After overnight transfection, the culture medium was replaced with phenol-free media supplemented with 10% FBS. Media was replaced 24 h post transfection on the A549/F, HT1080/F, HT1080-6TG/F, IIICF/c/F and MDA-MB-231 following which the cells were monitored over either 46 h or 60 h using the live cell time lapse imaging or were harvested at 48 h for western blot or fixed using 4% PFA for immunofluorescence. Three biological replicates were performed for the live cell imaging experiments and each experiment had at least three imaging fields.

### 4.4. Generation of YB-1:EBFP2 Expression Constructs

A two-stage process was used to create HA-tagged YB-1:EBFP2 expression constructs. Firstly an oligonucleotide encoding an HA tag flanked by XhoI and HindIII 5′ and SacII/BamHI 3′ was cloned into the XhoI/BglII sites in the MCS of pEBFP2-N1 plasmid (Addgene 54595, Addgene, Watertown, MA, USA) in-frame with EBFP2. The coding sequence of YB-1 and upstream Kozac sequence was amplified from an HA tagged YB-1 plasmid (pHA-YB-1) using TaKaRa Hi Fidelity Taq (Takara, Kyoto, Kyoto, Japan) and cloned upstream of the HA tag XhoI/HindIII. The resulting plasmid produced a YB-1 plasmid tagged with HA and a blue fluorescent protein EBFP2 (YB-1) fusion protein under the control of the CMV promoter. Unique restriction sites within the YB-1 sequence was used to replace wild-type sequence with sequence containing a serine to alanine mutation at S102, S165, S167, S174, S176 and S314. All plasmids were propagated through *Stbl3 Escherichia coli* to prevent recombination and sequenced prior to experimental use.

### 4.5. Plasmid Transfection

Cells (1 × 10^4^) were seeded in 2 mL of DMEM supplemented with 10% FBS in a 12 well plate. After overnight incubation, cells were transfected using Lipofectamine 3000 (LF3000, Invitrogen) with 250 ng of shRNA targeting the 3′UTR (sh-YB-1) in combination with 250 ng of either Vo:EBFP2 (Vo) or YB-1:EBFP2 (YB-1) or the six phospho-defective mutants (S102A:EBFP2–S102A, S165A:EBFP2–S165A, S167A:EBFP2–S167A, S174A:EBFP2–S174A, S176A:EBFP2–S176A and S314A:EBFP2–S314A). LF3000 (2.5 µL) was diluted in 100 µL of OptiMem, mixed and incubated for 5 min. In parallel, the plasmid was added to 100 µL of OptiMem (Invitrogen) along with 2.5 µL of P3000 and combined with the tube containing LF3000 and incubated for a further 10 min. 200 µL of the lipoplexes formed were added to the cells. Media was changed 24 h post transfection following which the cells were monitored over 60 h using the live cell time lapse imaging or were harvested at 48 h for Western blotting as described below. Live cell imaging experiments were performed three times. Western blot analysis was determined for each biological experiment containing three technical replicates.

### 4.6. Kinase Inhibitor Treatment and Cell Cycle

A549/F (1 × 10^4^) cells were seeded in 1 mL of DMEM supplemented with 10% FBS, 1% NEAA and 1% GlutaMax in a 24 well plate. After overnight incubation media was replaced with media containing either 0.01%(*v*/*v*) DMSO or kinase inhibitors as follows: 10 nM–1 µM Barasertib (AURKB), 0.1–5 µM BI-D1870 (RSK), 0.1–100 µM MK-2206 HCl (AKT) and 0.5–50 µM Silmitasertib (CSK2). Kinase inhibitors were obtained from and stock solutions were made up and stored as per manufacturer’s instruction (Selleckchem, Houston, TX, USA). Confluence was used to measure cell growth by imaging four fields per well at 2 h intervals for 48 h using the IncuCyte FLR and accompanying software (Essen BioScience, Ann Arbor, MI, USA). This experiment was performed twice.

A549/F cells were also seeded (1 × 10^4^) into 2 mL of DMEM supplemented with 10% FBS, 1% NEAA and 1% GlutaMax in a 12-well plate for live cell time lapse imaging and for western blot. Cells were concurrently seeded (5 × 10^3^) on glass coverslips in 24 well plates for immunofluorescence. After overnight incubation, DMEM was replaced with medium containing either 0.01% (*v*/*v*) DMSO or 1 µM Barasertib (AURKB inhibitor), 5 µM BI-D1870 (RSK inhibitor), 10 µM MK-2206 HCl (AKT inhibitor) and 10 µM Silmitasertib (CSK2 inhibitor). Cells were then either tracked for 46 h using the live cell time lapse imaging or were fixed at 37 °C for 12 min with 4% phosphate buffered paraformaldehyde (PFA) at time intervals between 18 and 28 h following treatment for immunofluorescence. Live cell imaging experiment was performed twice. There were at least three biological replicates and three technical replicates for each immunofluorescence experiment.

### 4.7. Cell Extracts and Western Blot

A549/F, HT1080/F, HT1080-6TG/F, IIICF/c/F and MDA-MB-231 (1 × 10^4^) cells were transiently transfected with either 5 nM si-Ctrl, 5 nM si-YB-1 a combination of si-YB-1 and si-p53, or Vo or sh-YB-1 as described above. In another experiment, A549 cells were treated with either DMSO or inhibitors of RSK and AURKB. 48 h post transfection or drug treatment, the media was discarded and the wells were washed with PBS and frozen at –80 °C. Cells were lysed in RIPA lysis buffer (150 mM sodium chloride, 1.0% NP-40 or Triton X-100, 0.5% sodium deoxycholate, 0.1% SDS (sodium dodecyl sulfate), 50 mM Tris, pH 8.0). Zebrafish embryos were dechorionated and deyolked prior to lysis. Total protein concentration was determined using the Pierce BCA kit (ThermoFisher Scientific, Waltham, MA, USA). Proteins were separated by electrophoresis on Bolt™ 4–12% Bis-Tris Plus Gels (Invitrogen). Proteins were transferred onto nitrocellulose membranes using iBlot gel transfer stacks (Invitrogen). Membranes were blocked with Odyssey^®^ Blocking Buffer (PBS) (LiCor, Lincoln, NE, USA) for 1 h and then incubated for 1 h with the primary antibodies (rabbit anti-YB-1 [41] at 1:1000, and mouse anti-β-actin at 1:10,000) diluted in Odyssey^®^ Blocking Buffer (PBS)/0.2% Tween 20 and then incubated for 1 h with the secondary antibodies (IRDye^®^ 680RD Goat anti-Mouse IgG (LiCor) and IRDye^®^ 800CW Goat anti-Rabbit IgG (LiCor)) diluted in Odyssey^®^ Blocking Buffer (PBS)/0.2% Tween 20. Membranes were imaged on the Odyssey^®^ CLx Imaging System (LiCor) according to the manufacturer’s instructions. Images were quantified using Image Studio (LiCor) software.

### 4.8. Live Imaging and Processing of Time-lapse Data

For live cell imaging, cells were grown in 12-well glass bottom plates (MatTek, Ashland, MA, USA). Time lapse live cell imaging was performed on a ZEISS Cell Observer inverted wide field microscope, with 20 × 0.8 NA air objective, at 37 °C, 10% CO_2_ and atmospheric oxygen. Images were captured every six minutes for a duration of sixty hours using an Axiocam 506 monochromatic camera (Zeiss) and Zen software (Zeiss). A Zeiss HXP 120 C mercury short-arc lamp and compatible filter cubes were used to obtain fluorescent images and differential interference contrast (DIC) microscopy to capture brightfield images. To achieve optimal image resolution without excessive illumination a binning factor was applied prior to imaging and the ambient conditions were maintained to minimize variations in optical resolution and illumination. The acquired videos were analysed using the Zen Blue software (Zeiss). For all videos, mitotic duration and outcomes were scored by eye and calculated from nuclear envelope breakdown until cytokinesis. FUCCI videos were scored by eye, for G_1_ (red) and S-G_2_-M (green). During time lapse imaging, some cells migrated away from the field of view, so the fate of those cells could not be determined and they were removed from further analyses.

Time lapse live cell imaging was also carried out on MDA-MB-231 cells that were transiently transfected with siRNA, seeded into a 2-well glass bottom µ-slide (ibidi, Planegg, Germany) and subsequently cultured in a heated and humidified chamber (ibidi) under cell culture conditions (37 °C, 5% CO_2_). Images were acquired every 15 min for 48 h following transfection using a Nikon Ti-E inverted microscope with a 20 × 0.75 NA dry objective and equipped with a Nikon DSRi2 CCD camera. The acquired images were analysed with NIS elements (Nikon, Minato City, Tokyo, Japan) and ImageJ software (Maryland, VA, USA). Live cell imaging experiments were performed twice.

### 4.9. Cell Synchronization

A549 (1 × 10^4^) cells were seeded on 13 mm diameter coverslips in a 24-well plate. After overnight incubation, cells were treated with 2 mM thymidine (Sigma-Aldrich) for 18 h, washed twice with PBS and released into medium without thymidine for 7–8 h. The cells were again treated with 2 mM thymidine (Sigma-Aldrich) for 15–16 h, washed twice with PBS and incubated in medium without thymidine for 6–7 h following which cells were fixed in 4% phosphate buffered paraformaldehyde (4% PFA) for 12 min and then washed thoroughly with PBS. There were six biological replicates with three technical replicates for each synchronization experiment.

### 4.10. Cell Proliferation Assays

A549 (2 × 10^3^) cells were seeded in 80 µL of DMEM supplemented with 10% FBS in a 96-well plate. After overnight incubation, 80 µL of media containing either 0.01% (*v*/*v*) DMSO or 1 µM Barasertib (AURKB inhibitor) or 5 µM BI-D1870 (RSK inhibitor) was added to the cells. In parallel, cells were also transfected with 25 ng of either Vo or YB-1:EBFP2 using Lipofectamine 3000 (LF3000, Invitrogen) as described above. Each treatment had six biological replicates. Twenty microliters of the lipoplexes were added to the cells. At the indicated time points the medium was removed and the plates were frozen at –80 °C. After all the time points had been collected the plates were thawed and the DNA content measured using a SYBR Green I-based fluorimetric assay as described previously [74]. Briefly, 100 µL of lysis buffer (10 mM Tris-HCl pH 8.0, 2.5 mM EDTA and 1% (*v*/*v*) Triton X-100) containing 1:4000 SYBR Green I (Invitrogen) was added to the wells and the plates were incubated overnight at 4 °C. The plates were mixed and the fluorescence signal for each well was measured for 1 s at an excitation of 485 nm and emission of 535 nm using a BioTek microplate reader (BioTek, Winooski, VT, USA). Growth curves were plotted as the fluorescence values at each time point. There were at least three biological replicates for the control and the treated samples.

### 4.11. Immunofluorescence

Fixed cells were thoroughly washed in PBS, quenched with 0.1 M glycine in PBS, permeabilized with 0.2% Triton X-100 (Sigma-Aldrich) and following further washing with PBS, were blocked with 1% BSA in PBS with 0.2% cold fish skin gelatin (Sigma-Aldrich) prior to incubation with primary antibodies in blocking solution. Following primary incubation, cells were again washed thoroughly to remove any unbound primary antibody and then incubated with a fluorophore-labelled secondary antibody. Sequential immunolabelling was carried out in some instances, whereby cells were thoroughly washed with 0.1%TWEEN–20 (Sigma-Aldrich) in PBS and re-blocked prior to incubation with another primary. Filamentous (F)-actin was labelled with Phalloidin, 1:2000 (ThermoFisher Scientific). The DNA-binding dye Hoescht 33258, 1:2000 (Molecular Probes, Eugene, OR, USA) was used to visualise cell nuclei. Cells were thoroughly washed in PBS prior to mounting in Fluoromount-G (SouthernBiotech, Birmingham, AL, USA) on glass slides. Cells were imaged on a Nikon A1 inverted confocal microscope using a 60× oil-immersed objective.

The following primary antibodies were used: rabbit anti-YB-1 polyclonal to N-terminal (generated in house [41] 1:1000). Sheep anti-YB-1 polyclonal to N-terminal (generated in house). Mouse anti-AURKB (AIM-1, BD Bioscience Cat #611082, RRID: AB_2227708, 1:200, (BD transduction laboratories, Franklin Lakes, NJ, USA). Mouse anti-α-Tubulin (DM1A, Cell Signaling Technology Cat. #: 3873, RRID:AB_1904178, 1:2000, (Cell Signalling Technology, Danvers, MA, USA). Mouse anti-CAPZA1 (Proteintech, cat. #: 66066-1-Ig, 1:200, (Proteintech, Rosemont, IL, USA)). Mouse anti-Moesin (Novus Biologicals, cat. #: MSN/491, 1:200 (Novus Biologicals, Littleton, CO, USA). Rabbit anti-phospho-YB1^ser102^(pYB-1, Cell Signalling Technology, cat. #: C34A2, 1:1000). Rabbit anti-Histone H3 (D1H2, Cell Signaling Technology, cat. #: 4499, RRID:AB_10544537). Secondary antibodies used were goat anti-rabbit Alexa Fluor 488 (Molecular Probes, cat. #: A-11070, RRID:AB_142134, 1:1000), F(ab’)2 goat anti-mouse Alexa Fluor 350 (Molecular Probes, cat. #: A-11045, RRID:AB_142754, 1:1000) and F(ab’)2 goat anti-mouse Alexa Fluor 633 (Molecular Probes, cat. #: A-21052, RRID:AB_2535719, 1:1000).

### 4.12. Zebrafish Lines

Wild-type WIK zebrafish (ZFIN ID: ZDB-GENO-010531-2) were maintained as described previously [75]. Stages were determined by using both time since fertilisation (hpf) and morphological features [76]. All experimental procedures performed were in compliance with local animal welfare regulations and assessed by the Animal Ethics Committee (University of Otago, Dunedin, NZ).

### 4.13. Microinjection of Zebrafish Embryos

Antisense morpholino oligonucleotides were obtained from GeneTools LLC (Philomath, OR, USA). For microinjection, 1 nL of morpholino solution (4 µg/µL) diluted in Danieau’s buffer was injected into the yolk of embryos at the 1 to 2-cell stage. Morpholino sequences were MO_*ybx1* (morphilino targeting ybx1) 5′-GTTGTGTCTCGGCCTCGCTGCTCAT-3′ and MO_Ctrl (control morpholino), 3′-TAATTTACTTACCCTCAAGTTGCTG-5′. The images of the zebrafish embryos after treatment were taken using the 10× objective of the SMZ1500 stereoscopic zoom microscope (Nikon, Minato City, Tokyo, Japan).

### 4.14. Whole-Mount Immunofluorescence of Zebrafish Embryos

Whole-mount immunofluorescence was carried out according to a standard protocol, as previously described [77], In brief, embryos were fixed in 4% PFA in PBS at 4 °C overnight, dechorionated and stored in methanol at −20 °C. Following sequential rehydration into PBS with 0.1% Tween (PBSTw), embryos were immersed overnight in 30% sucrose at 4 °C before incubation at 70 °C in 150 mM Tris-HCl (pH 9.0). Following thorough washing with PBSTw, they were then blocked with 10% sheep serum, 0.8% Triton, 1% BSA in PBSTw for 3 h at 4 °C before incubation with primary antibodies in 1% sheep serum, 0.8% Triton, 1% BSA in PBSTw for 3 days at 4 °C on a rotating mixer. Residual primary antibody was then removed by successive 1hr washes in PBS with 10% sheep serum and 1% Triton before incubation in secondary antibodies and Hoechst dye in the dark for 2.5 days. Following removal of residual secondary antibodies by successive 1 h washes in PBS with 10% sheep serum and 1% Triton, embryos were mounted in Fluoromount-G (SouthernBiotech, AL, USA) and imaged on a Nikon A1 inverted confocal microscope using a 60× oil-immersed objective.. Antibodies used were rabbit anti-YB-1 polyclonal to N-terminal (generated in house [41] 1:1000), mouse anti-α-Tubulin (DM1A, Cell Signaling Technology, cat. #: 3873, RRID:AB_1904178, 1:500). Secondary antibodies used were F(ab’)_2_ goat anti-rabbit Alexa Fluor 568 (Molecular Probes, cat. #: A-11011, RRID:AB_143157, 1:1000), goat anti-mouse Alexa Fluor 488 (Molecular Probes, cat. #: A-11070, RRID:AB_142134, 1:1000).

### 4.15. Subcellular Fractionation of Cultured Cells

Cells were enriched into subcellular fractions as described previously [41]. Briefly, 1 × 10^4^ cells per µL were swelled in a hypotonic buffer (10 mM HEPES, pH 7.9, 1.5 mM MgCl_2_, 10 mM KCl, 0.5 mM, DTT, 1 × Complete EDTA-free), and incubated for 5 min at 4 °C before being transferred to a Dounce homogeniser (Kontes (Thomas Scientific, Swedesboro, NJ, USA), 885300-0002, 2 mL, tight pestle; 0.013–0.064 mm clearance). The Dounce was applied until 95% of the cell membranes were ruptured and lysate was spun at 220 g for 5 min at 4 °C. The supernatant was collected and stored as cytoplasmic extract. The pelleted nuclei were re-suspended in 5 mL of chilled low sucrose solution (0.25 M Sucrose, 10 mM MgCl_2_) which was gently layered over 5 mL of chilled high sucrose solution (0.88 M Sucrose, 10 mM MgCl_2_). The nuclei were spun at 2800 g for 10 min at 4 °C to remove contaminating cytoplasmic proteins.

### 4.16. Immunopurification of YB-1

YB-1 was immunopurified as described previously by Cohen et al. [42]. Lysates were prepared at the equivalent of 1 × 10^4^ cells per µL for whole cells or cytoplasmic lysates and 5 × 10^4^ cells per µL for nuclear lysates in a low detergent RIPA buffer (50 mM Tris-HCL, 150 mM NaCl, 0.5% NP40, 0.1% deoxycholate). Complete lysis of nuclei was ensured by passing the nuclei pellets and lysis buffer through a 22 gauge needle 10 times followed by incubation for 20 min at 4 °C. Debris was cleared from lysates by centrifugation at 13,000× *g* for 10 min. All lysates included protease and phosphatase inhibitors at the recommended concentration (Complete-EDTA-free and PhosSTOP (Roche, Basel, Switzerland)).

Protein G dynabeads were prepared for use following the manufacturer’s instructions (Life Technologies). Protein G dynabeads were immobilized on a magnet and washed three times with 300 µL of low-detergent RIPA buffer. 100 µg of anti-YB-1 was added to the lysates and incubated with gentle agitation for 1 hr at 4 °C. Twenty-five µL of protein G beads were then added for every 20 µg of antibody and incubated for another 30 min at 4 °C. The bead:antibody:YB-1 complexes were then immobilized by a magnet, the lysate removed, and the interaction of YB-1 and the anti-NYB1 was disrupted by the addition of the peptide to which NYB1 was raised at a concentration of 100-fold compared to the antibody. The immunopurified proteins were then stored at −80 °C to await further analysis.

### 4.17. Immunopurification of Cross-Linked YB-1

Proteins were cross-linked in vivo by treating cells with dithiobis[succinimidyl] propionate (DSP) buffered in PBS. Cells were trypsinised, counted, and rinsed twice with PBS. Immediately prior to its use, a 25 mM DSP solution was prepared in DMSO ([CH_3_]_2_SO). The cells were diluted to 1 × 10^4^ cells per µL in PBS and the 25 mM DSP solution was added to the cell suspension at a ratio of 1:25. Cross-linking took place with gentle mixing at room-temperature for 45 min. The cells were spun and the supernatant removed. The remaining cross-linker was then quenched by the addition of 100 mM Tris in PBS which was added in a stoichiometry of at least 1:1 with the cross-linking reagent dithiobis-succinimidyl propionate (DSP) and incubated at room temperature for 10 min. This step was repeated once before the cells were lysed at 1 × 10^4^ cells per µL in low-detergent RIPA, snap frozen, and stored at −20 °C. The Protein G Mag Sepharose beads were used at a concentration of 75 µL for every 50 µg sheep-anti-YB-1. For these IPs, the antibody and Protein G Mag Sepharose beads were complexed together in 500 µL of low-detergent RIPA for 30 min, immobilized, and then rinsed once with another 500 µL of soft buffer. The lysate was then added to the immobilized antibody plus Protein G Mag Sepharose beads and rotated for 1 h at 4 °C. The interaction of the antibody with the protein G beads was disrupted using competitive elution. The peptide to which NYB1 was raised was added to each sample at a concentration of 100-fold to native PAGE buffer (Life Technologies) with 1% Digitonin (Sigma), 1 × phosSTOP (Roche) and 1 × complete EDTA- (Roche). The samples were incubated at room temperature for 2 h with gentle mixing. Following this, the samples were concentrated using centrifugation at 15,000 g at room temperature in a Vivaspin 500, 30 000 MWCO column (Sartorius) until their volume was 25 µL.

### 4.18. Mass Spectrometry

LC-MS/MS was used to identify the proteins that copurified with immunoprecipitated YB-1. The immunopurified proteins were separated using SDS-PAGE and the gel was stained using colloidal coomassie staining (34% methanol, 17% (*w*/*v*) (NH_4_)_2_SO_4_, 3% phosphoric acid, 0.1% Coomassie G-250 (BioRad, Hercules, CA, USA)). Protein bands and fractions were excised and subjected to in-gel digestion with trypsin using the automated in-gel digestion method [78]. For the identification of sites of phosphorylation, tryptic fragments from the digested YB-1 bands were subjected to phosphopeptide enrichment using TiO_2_ affinity chromatography [79]. All samples were further purified by solid-phase extraction on C18 material.

For targeted detection of phosphorylated YB-1 peptides, phospho-enriched peptide fractions were prepared from whole cells. Peptides were generated from 500 µg of protein from whole cell lysates by tryptic protein digestion using the filter-aided sample preparation method [80]. Phosphorylated peptides were enriched from 450 µg of digested protein peptides using a TiO_2_/ZiO_2_ TopTip following the manufacturer’s protocol (TT2TIZR, Glygen, Columbia, MD, USA).

All samples except those for targeted sequencing were re-solubilized in 1% (*v*/*v*) acetonitrile, 0.2% (*v*/*v*) formic acid in ultrapure H_2_O, and analysed on three different LC-coupled linear ion trap/orbitrap instruments located at the Centre for Protein Research (Dunedin, Otago, NZ), the Children’s Medical Research Institute (Sydney, NSW, Australia), and the Bioanalytical Mass Spectrometry Facility (NSW, Australia). For targeted analysis of YB-1 phosphorylation enriched phosphorylated peptides for targeted analysis of YB-1 phosphorylation were re-solubilised in 2% (*v*/*v*) acetonitrile, 0.2% (*v*/*v*) formic acid in ultrapure H_2_O and separated using an LC-coupled TripleTOF 5600+ (AB SCIEX, Framingham, MA, USA). The method used at the Centre for Protein Research is here described as a representative instrument method. Peptides were separated on emitter-tip columns (75 µm ID fused silica tubing packed with C-18 material on a length of 12 cm). The gradient for liquid chromatography was modified depending on the complexity of the fraction or band. Generally, the gradient developed from 1% (*v*/*v*) acetonitrile, 0.2% (*v*/*v*) formic acid to 80% (*v*/*v*) acetonitrile, 0.2% (*v*/*v*) formic acid in ultrapure H_2_O at a flow rate of 400 nL/min.

Full MS (MS1) in a mass range between *m/z* 400–2000 was performed in the Orbitrap mass analyser with a resolution of 60,000 at *m/z* 400 and an AGC target of 2 × 10^5^. The five strongest signals, with a charge state ≥ [M + 2H]2+, were selected for collision induced dissociation -MS/MS (MS2) in the LTQ ion trap at a normalized collision energy of 35% using an AGC target of 1 × 10^5^ and two microscans. Dynamic exclusion was enabled with two repeat counts during 30 s (sec) and an exclusion period of 180 s. The exclusion mass width was set to 0.01.

Peaklists and MS/MS data were generated using the Proteome Discoverer (version 1.4) software using default settings and searched against the Human Swiss-Prot amino acid sequence database using both the Mascot (http://www.matrixscience.com) and Sequest (ThermoFisher Scientific) search engines. The search was set up for full tryptic peptides with a maximum of three missed cleavage sites. Carboxyamidomethyl cysteine, oxidized methionine, deamidation (N, Q), and phosphorylation (S, T, Y) were included as variable modifications. The CAMthiopropanoyl (K) modification, which corresponds to a DSP adduct that has been reduced and alkylated, was added to searches of LC-MS/MS data from cross-linked samples. The precursor mass tolerance threshold was 10 ppm and the maximum fragment mass error 0.8 Da. Peptides were accepted as identified if their false discovery rate adjusted scores were above the score threshold at a false discovery rate of ≥ 1%, as assessed by the Percolator algorithm [81]. Proteins were considered identified when they were assigned ≥ 2 peptides above the aforementioned score threshold.

Phosphorylated YB-1 peptides using targeted mass were detected using multi-reaction monitoring mode where the precursor *m/z* of phosphorylated YB-1 peptides that were detected in immunopurified YB-1 samples were subjected to MS2 scans throughout the gradient. Levels of YB-1 in the enriched phosphorylated samples were also estimated by using a similar gradient and sequential window acquisition of all theoretical fragment ion spectra mass spectrometry (SWATH) method on a Triple TOF 5600+ mass spectrometer. The data from targeted LC-MS/MS and SWATH were analysed using Mascot (http://www.matrixscience.com) and Skyline (v4.2.0). Quantification of phosphorylation at different sites on YB-1 was performed using the peak areas from fragment ions unique to individual phosphorylation sites.

### 4.19. Modeling the Structure of YB-1 Protein

The automated I-TASSER pipeline [65] which generates atomic models of proteins based on multi-threading alignment and iterative template fragment assembly simulations was used to model the structure of full length YB-1. This yielded several models of which we chose the model that incorporated the coordinates of the CSD of YB-1 whose three-dimensional structure has been resolved by NMR [66] and was one of the templates used by I-TASSER.

### 4.20. Molecular Dynamics Simulations

The three-dimensional model of full length YB-1 was subject to molecular dynamics (MD) simulations in its phosphorylated form (at Serines 102, 165, 167, 174, 176 and 314, referred to as pYB-1), unphosphorylated form and of mutant forms, S167A and S176A (where the two mutant forms had the other 5 serines phosphorylated) using the *pmemd.CUDA* module of the program Amber14 [82]. The all atom version of the Amber 14SB force field (ff14SB) [83] was used for the protein. Force field parameters for phosphorylated serines were taken as described elsewhere [84]. For the phosphate groups, an overall charge of −2 e was used. The *Xleap* module of Amber14 was used to prepare the system for the MD simulations. All the simulation systems were neutralized with appropriate numbers of counterions. Each neutralized system was solvated in an octahedral box with TIP3P [85] water molecules, leaving at least 10 Å between the solute atoms and the borders of the box. All MD simulations were carried out at 300 K. During the simulations, the long-range electrostatic interactions were treated with the particle mesh Ewald [86] method using a real space cutoff distance of 9 Å. The Settle [87] algorithm was used to constrain bond vibrations involving hydrogen atoms, which allowed a time step of 2 fs during the simulations. Solvent molecules and counterions were initially relaxed using energy minimization with restraints on the protein. This was followed by unrestrained energy minimization to remove any steric clashes. Subsequently the system was gradually heated from 0 to 300 K using MD simulations with positional restraints (force constant: 50 kcal mol^−1^ Å^−2^) on the protein over a period of 0.25 ns allowing water molecules and ions to move freely. During an additional 0.25 ns, the positional restraints were gradually reduced followed by a 2 ns unrestrained MD simulation to equilibrate all the atoms. For each system, three independent MD simulations (assigning different initial velocities) were carried out for 250 ns with conformations saved every 10 ps. Simulation trajectories were visualized using VMD [88] and figures were generated using Pymol [89].

### 4.21. Statistical Analysis

One tailed Student’s t-test was performed to assess the significant differences between control and treated cells. Mann-Whitney U test was used to determine the significance of the difference in distribution between cell cycle phases of control and treated cells. Chi-square and Fisher’s Exact test was used to assess significant differences in the distribution of number of twisted furrows between control and YB-1 depleted cells. One-way ANOVA with Tukey’s multiple comparison test was used to assess the significance of difference in percentage of multinucleate cells in RSK and AURKB treated cells with and without YB-1 overexpression. All statistical analyses were performed using GraphPad Prism software version 7.03 (SD, USA).

## 5. Conclusions

In summary, we define precisely how YB-1 regulates cell division. We provide unequivocal evidence that defects in YB-1 result in cytokinesis failure and multinucleation in cancer cells. We demonstrate that YB-1 is essential for microtubule organization and facilitates the specification of the cleavage plane in vitro and in vivo, thus establishing its physiological function. We show that RSK phosphorylates multiple sites on YB-1 and highlight the importance of the YB-1/RSK axis during cytokinesis. Finally, using molecular modelling we demonstrate the importance of structural rearrangement regulated by simultaneous phosphorylation for YB-1 function during cytokinesis. Our work conclusively shows phosphorylated YB-1 is critical for assembly of microtubules, specification of the cleavage plane and localization of the CPC during cytokinesis.

## Figures and Tables

**Table 1:** Summary of YB-1 phosphorylation sites identified using global LC-MS/MS profiling in A549, MDA-MB-231 and T47D cells. Numerator: number of times phosphorylation site was detected; denominator: total number of LC-MS/MS runs.

Amino Acid	MDA-MB231		A549		T47D	
	Nucleus	Cytoplasm	Nucleus	Cytoplasm	Nucleus	Cytoplasm
S102			3/8	3/8		
S165	2/2	2/2	5/8	5/8	1/1	1/1
S167	1/2		3/8	3/8	1/1	1/1
S174		1/2	3/8	4/8	1/1	1/1
S176		1/2	4/8	4/8	1/1	1/1
S314	1/2	2/2	5/8	5/8	1/1	1/1
S03			1/8	2/8		
T07			1/8	1/8		
S21			3/8			
S209			4/8	3/8		
T271			2/8			

## Supplementary Materials

The following are available online at https://www.mdpi.com/2072-6694/12/9/2473/s1, Figure S1. Western blots; Figure S2. YB-1 protein partners identified using LC-MS/MS. Figure S3a: YB-1 depletion decreases number of cell divisions; Figure S3b: Dose dependent decrease in growth of A549/F cells treated with kinase inhibitors; Figure S4a. Representative MS/MS spectra showing phosphorylation of YB-1 at residues S165, S174, S176 and S314; Figure S4b. RSK inhibition prevents phosphorylation of YB-1 at multiple serines; Figure S4c. EBFP2 constructs expressed similar levels of protein; Video S1. A549/F cells treated si-Ctrl; Video S2. A549/F cells treated with si-YB-1.
