# Supplementary material for: Critical Role for Cold Shock Protein YB-1 in Cytokinesis
Source: Cancers (Basel). 2020 Sep 1;12(9):2473. doi: 10.3390/cancers12092473 (PMC7565962; doi:10.3390/cancers12092473)
Supplement: Supplementary file 1 [file cancers-12-02473-s001.pdf]

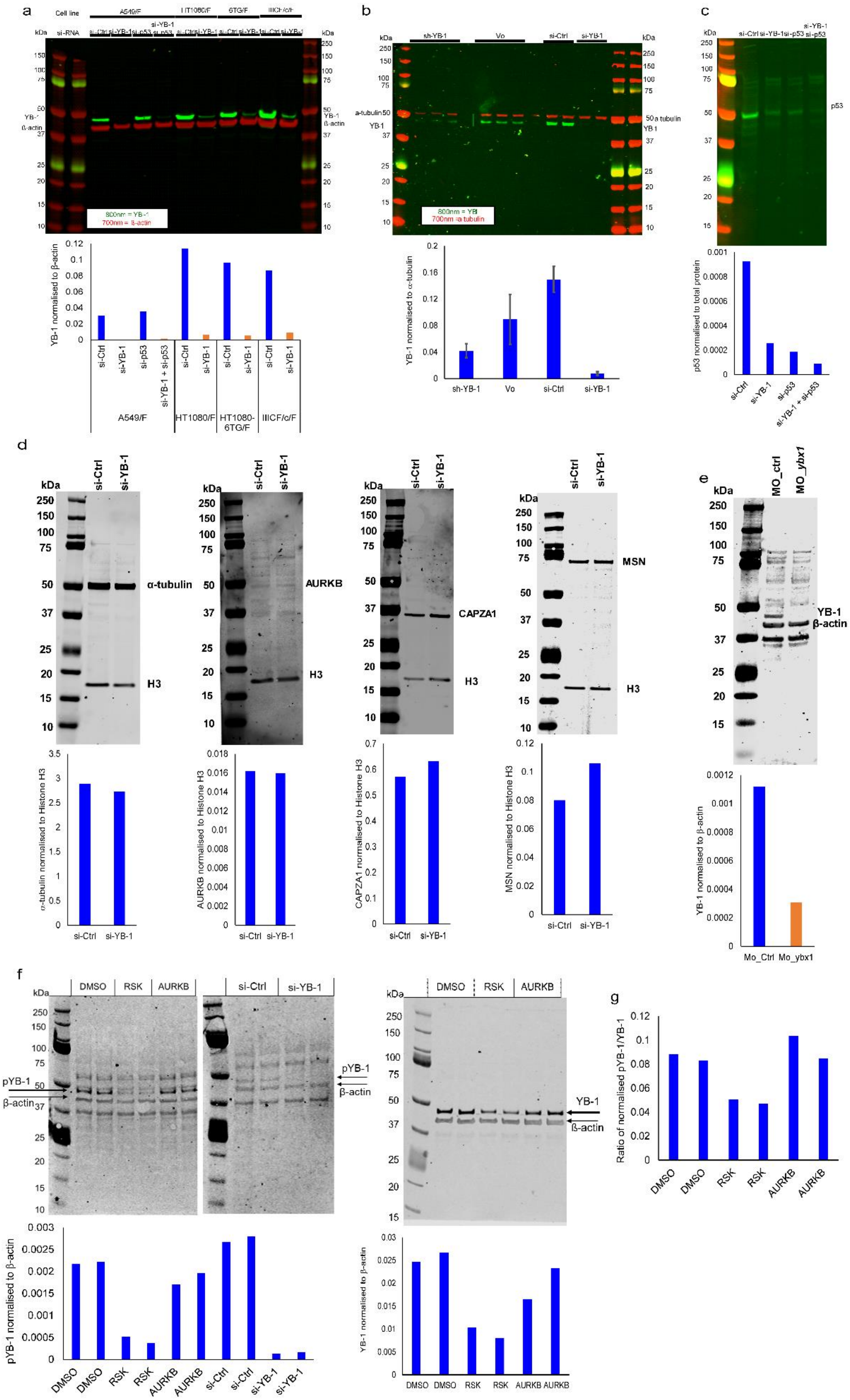

a
Cell line
A549/F
HT1080/F
6TG/F
IIICF/c/F
si-RNA
si-YB-1
800nm = YB-1
700nm = β-actin
YB-1
β-actin
YB-1 normalised to β-actin
si-Ctrl
si-YB-1
si-p53
si-YB-1 + si-p53
A549/F
HT1080/F
HT1080-6TG/F
IIICF/c/F
b
sh-YB-1
Vo
si-Ctrl
si-YB-1
α-tubulin
YB-1
800nm = YB
700nm = α-tubulin
YB-1 normalised to α-tubulin
c
si-Ctrl
si-YB-1
si-p53
si-YB-1 + si-p53
p53
p53 normalised to total protein
d
kDa
α-tubulin
H3
AURKB
CAPZA1
MSN
α-tubulin normalised to Histone H3
AURKB normalised to Histone H3
CAPZA1 normalised to Histone H3
MSN normalised to Histone H3
e
MO_ctrl
MO_ybx1
YB-1
β-actin
YB-1 normalised to β-actin
Mo_Ctrl
Mo_ybx1
f
DMSO
RSK
AURKB
si-Ctrl
si-YB-1
pYB-1
β-actin
YB-1
pYB-1 normalised to β-actin
YB-1 normalised to β-actin
g
Ratio of normalised pYB-1/YB-1
DMSO
RSK
AURKB

Figure S1: Western Blots and quantitation. a. Western blot of YB-1 and β-actin protein after treating cells for 48h with either si-YB-1 (5nM) or si-Ctrl (5nM) in A549/F, HT1080/F, HT1080-6TG/F and IIICF/c/F cells. **b.** Western blot of YB-1 and α-tubulin protein after treating cells with 48h of either si-YB-1 (5nM) or si-Ctrl (5nM) in MDA-MB-231 cells or treating A549/F cells with either an sh-YB-1 targeting the 3'UTR of YB-1 (1μg) or Vo (1μg). Bars show mean ± s.d. from 3 replicates. **c.**Western Blot of p53 knockdown in A549/F cells after treating cells with either si-Ctrl, si-YB-1 or si-p53 for 48 hours. **d.** Western blot showing the levels of α-tubulin, AUKRB, CAPZA1, MSN and Histone H3 in A549/F cells treated for 48h with either si-YB-1 (5nM) or si-Ctrl (5nM). **e.** Western blot of *ybx1* and β-actin protein after treating zebrafish embryos with either MO_Ctrl or MO_*ybx1*. **f. Left:** Western blot of phosphorylated YB-1 at S102 (pYB-1) and β-actin in A549 cells treated with either DMSO, RSK inhibitor, AURKB inhibitor, si-Ctrl or si-YB-1. **Right:** Western blot of YB-1 and β-actin protein in cells treated with either DMSO, BI-D1870-5μM (RSK) or Barasertib-1μM (AURKB). g. Ratio of normalized pYB-1/YB-1 in A549 cells treated with either DMSO, RSK inhibitor, AURKB inhibitor.

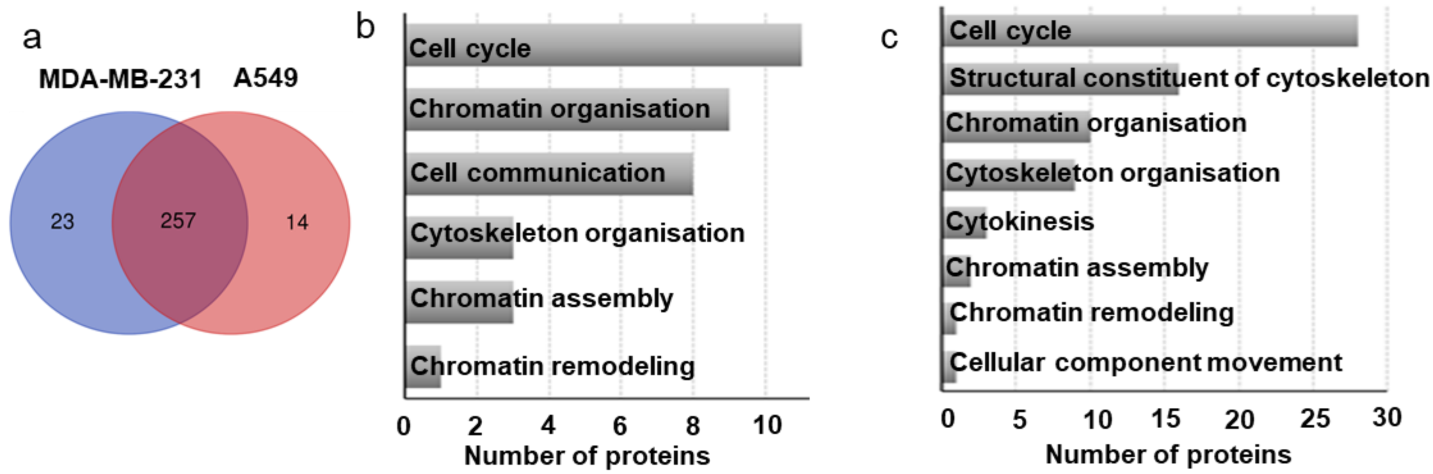

Figure S2: YB-1 protein partners identified using LC-MS/MS. a. The number of common protein partners immunoprecipitated with YB-1 in the two cell lines MDA-MB-231 and A549. **b.** Summary of the functional analyses on the common protein partner's from A549 and MDA-MB-231 immunoprecipitated with YB-1. **c.** Summary of the functional analyses on the cross-linked protein partner's immunoprecipitated with YB-1. b-c. Functional analyses was performed using Pantherdb.

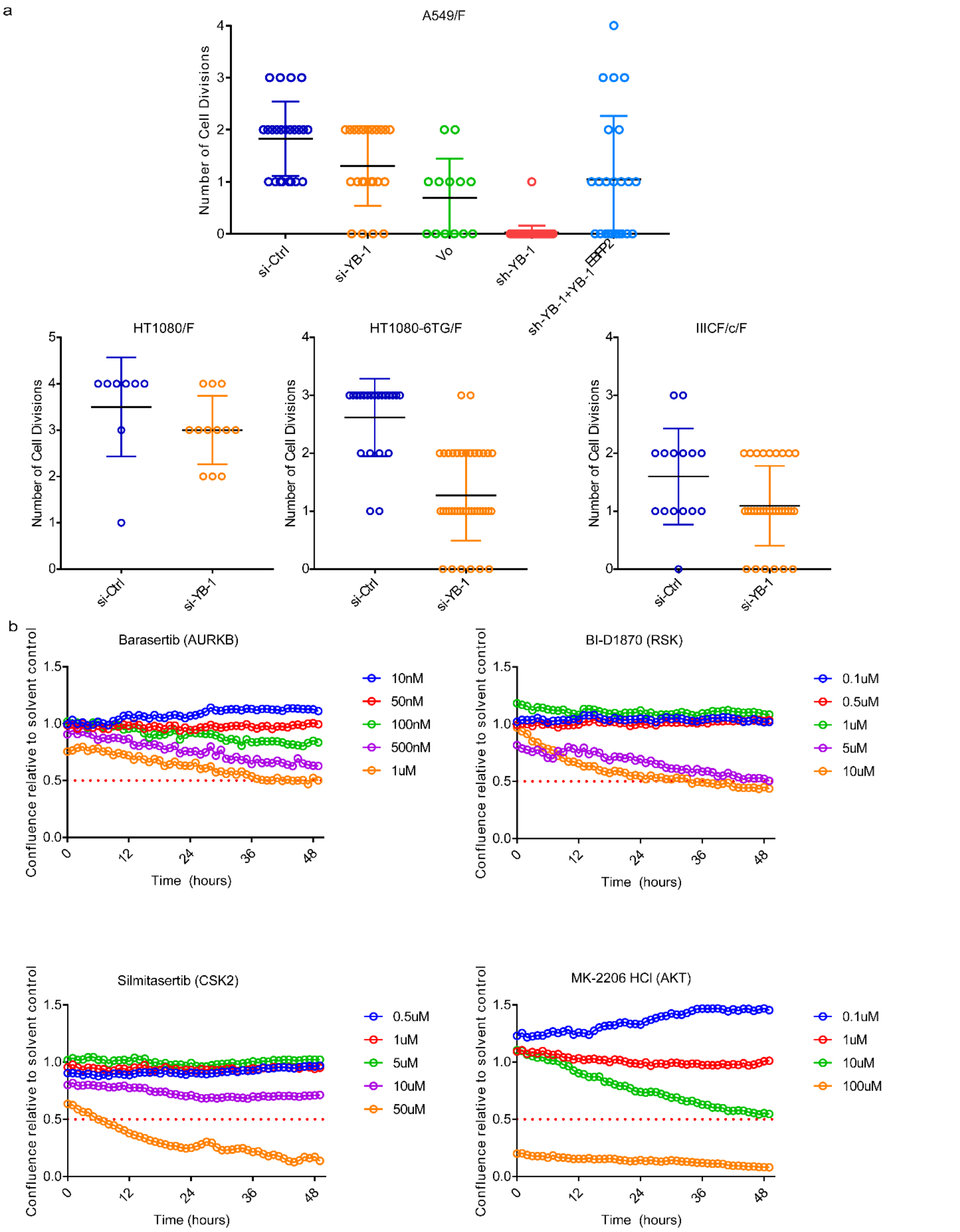

Figure S3: a. YB-1 depletion decreases number of cell divisions. Number of cell divisions of A549/F cells in cultures treated with si-Ctrl, si-YB-1, Vo, sh-YB-1 and sh-YB-1+YB-1^{BFP2} post

transfection (top panel).Number of cell divisions of HT1080/F, HT1080-6TG/F and IIICF/c/F cells treated with either si-Ctrl or si-YB-1 post transfection (bottom panel). The line in the middle of each box represents the median, the top and bottom outlines of the box represent the first and third quartiles. Significance was determined using Mann-Whitney U test, * $p < 0.05$, *** $p < 0.001$**.**
b. Dose dependent decrease in growth of A549/F cells treated with kinase inhibitors. Growth response of A549/F cells treated with: 10nM - 1µM Barasertib (AURKB - inhibitor), 0.1µM - 10µM BI-D1870 (RSK - inhibitor), 0.1µM - 100µM MK-2206 HCl (AKT - inhibitor) and 0.5µM - 50µM Silmitasertib (CK2 - inhibitor). Confluence was used to measure cell growth by imaging 4 fields per well at 2 hour intervals for 48h using the IncuCyte FLR and accompanying software.

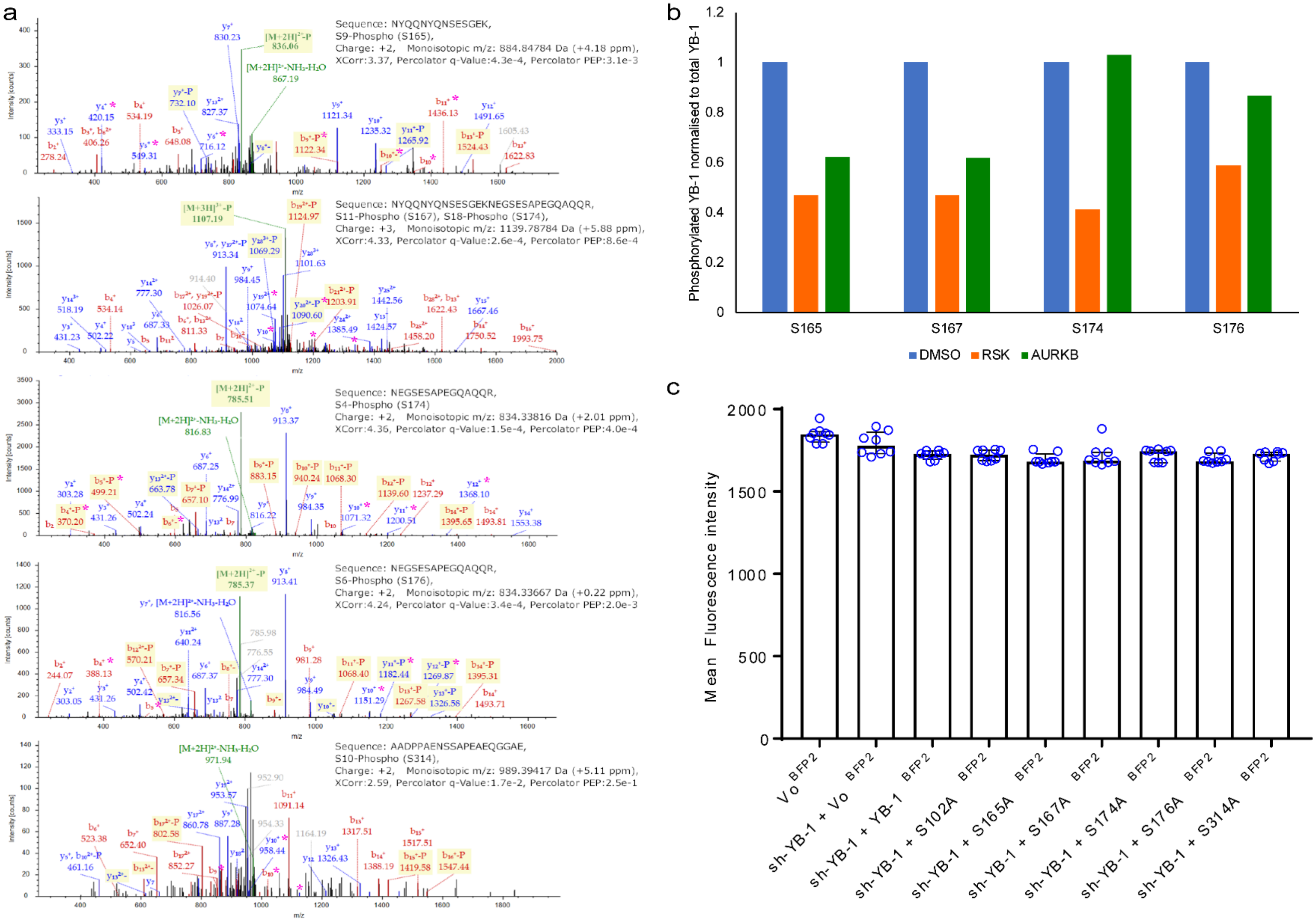

Figure S4: a. Representative MS/MS spectra showing phosphorylation of YB-1 at residues S165, S174, S176 and S314. The annotated peaks were identified as b-ions (red) and y-ions (blue) by Sequest. Phosphorylation was identified as the addition of HPO_3 (+ 79.96633 Da) or the neutral loss of H_3PO_4 (- 97.976896; annotated by "-P" and a yellow background). Ions produced by neutral losses from the precursor ion are shown in green. Peaks that clearly indicate the site of the phosphorylation are annotated with a "*". **b. RSK inhibition prevents phosphorylation of YB-1 at multiple serines determined using targeted LC-MS/MS**. Mass

spectrometry analyses of phosphorylated YB-1 at S165, S167, S174 and S176 in A549 cells treated with either DMSO, Barasertib-1µM (AURKB) or BI-D1870-5µM (RSK). **c. EBFP2 constructs expressed similar levels of protein.** The graph shows the average of the arithmetic mean intensity of the individual EBFP2 constructs determined by time lapse live cell imaging. The bars and error bars represent the mean ± s.d. across eight imaging fields.
